# Supplementary material for: Wealth and inequality gradients for the detection and control of hypertension in older individuals in middle-income economies around 2007-2015
Source: PLoS One. 2022 Jul 8;17(7):e0269118. doi: 10.1371/journal.pone.0269118 (PMC9269405; doi:10.1371/journal.pone.0269118)
Supplement: S1 Appendix — (PDF) [file pone.0269118.s001.pdf]

Wealth and inequality gradients for the detection and control of hypertension in older individuals in middle-income economies
Appendix

July 26, 2021

A Hypertension diagnosis and treatment in LMIC

Despite improvements in the last decades, treatment and control of hypertension vary widely in LMIC, not only between countries and communities but also between urban and rural areas (Prince et al., 2012). While there is a wide variety of health systems in place, from pure national health services to managed competition models, there are common elements to consider (Alshamsan et al., 2017). First, there are substantial differences in the quality of health services that individuals can access according to their health insurance. Hence, the possibility to access to voluntary health insurance (VHI) is reflected also in health inequalities. Second, disparities regarding availability of staff in urban and rural areas have played an essential role in the development of community-based models (CBM), where local staff receive basic training for dealing with primary health care. There are, however, significant differences concerning the specific needs and challenges of each country. Table A1 on the characteristics of the health systems, and each country profile below presents further details for each of them. We also include statistics in terms of the GDP per capita in 2020 USD dollars and the GINI concentration index, from the World Bank indicators.

Table A1: Health systems

	China	Colombia	Ghana	India	Mexico	Russia	South Africa
**Health system**							
Insurance structure	Mandatory public health insurance but which varies accordingto the location of residence of the individual	Compulsory Health Insurance. Contributory regime for the population with the ability to pay and a subsidized regime for the population without the ability to pay.	National health insurance system with a low yearly premium	Universal access to a free health insurance.	The services received by the population depend on individuals' employment status and ability to pay.	The "Semashko" centralized health-care system.	Universal access is guaranteed but under a separated private and public health systems.
Funding sources/Finantial coverage	Urban employer-based insurance: mainly employer/employee payroll taxes, with minimal government funding. Residency-based Basic Medical Insurance: Individual premiums, mostly funded by central and local government subsidies.	Tax on wages + general budget, out-of-pocket for supplementary HI	Taxes+health insurance contributions made up of premiums + out-of-pocket payments	Government contribution+ private insurance+ out-of-pocket payments	Federal contributions, state contributions and family contributions.	HI is totaly financed by government	Government, households, employers and Non-Governmental Organisations (NGOs).
Insurers		Both regimes operate with public and private insurers	Public and private insurers	Public and private insurers	Public and private insurers	Public Insurers	Public and private insurers
**Health coverage**							
UHC index of service coverage (SCI) (2017)	79	76	47	55	76	75	69
UHC SCI components: Noncommunicable diseases (2017)	65	77	79	64	72	56	58
UHC SCI components: Service capacity and access (2017)	100	85	31	46	80	100	80
Median availability of selected generic medicines (%) - Private	13.3	90.7		2.8	50	100	
Median availability of selected generic medicines (%) - Public	15.5	86.7			42.6	100	

Notes: Universal Health Coverage indicators obtained from WHO

Figure A1: Densities of the systolic BP by country

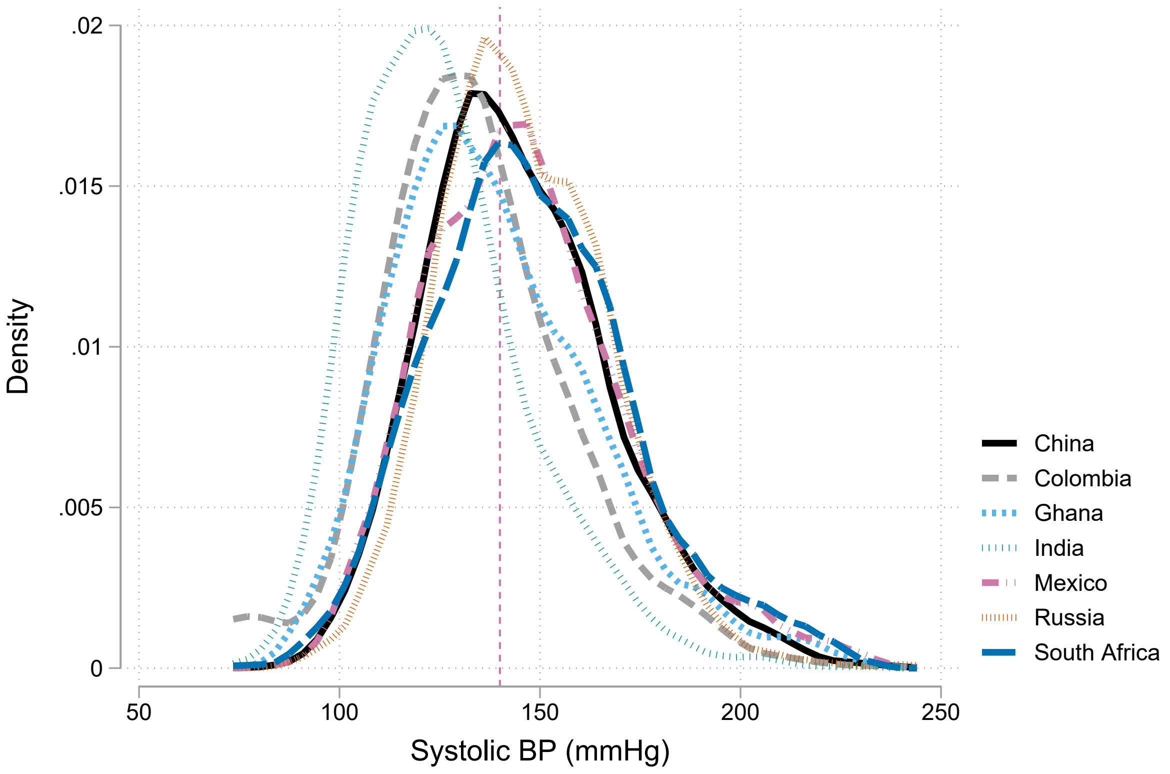

Notes: Epanechnikov Kernel densities using a bandwidth of 4 mmHg. Vertical line corresponds to the standard 140 mmHg threshold for diagnosis. Graph constructed over the sample of SABE and SAGE of respondents with a valid BP measure and a self-report of HBP.

A.1 China

In China, the general population is covered by mandatory public health insurance which varies between rural and urban areas (Fang and Commonwealth Fund, 2017). However, there exist deductibles and co-payments, which are reflected in the level of out-of-pocket expenditures. There is access to VHI markets for high-income individuals to complement or replace the public system. Citizens can access their local doctor or directly to any GP in a hospital. Hypertension in China is an important public health issue. In particular, Wang (2019) highlights that there is a high risk due to too high sodium intake, which also affects its pathology.

By 2012/15, around 23% of the population had HBP, 47% of them are aware of the condition, and 15% of them had it under control Wang et al. (2018). Huang et al. (2016) argues that China's issue is mainly about detection rather than treatment. Their meta-study shows that access to health insurance is a crucial factor for awareness and correct management. In particular, they highlight the critical differences between those with private voluntary insurance and those with the mandatory insurance (Feng et al., 2013; Guo et al., 2015; Basu and Millett, 2013). Other clear improvement areas are access and the implementation of guidelines, where the CBM model presents promising results (Li et al., 2015; Niu et al., 2014; Zheng et al., 2019).

In terms of income, China had a GDP per capita of around 2.793 USD by 2007 and of 4.550 USD by 2010 (fifth in the study group). The GINI coeficient for the country was around 43 in those years (fourth).

A.2 Colombia

The Colombian health system provides almost universal health insurance to its nationals, where each individual has to be affiliated to a health maintenance organisation (*Entidades Promotoras de Salud*, EPS) which receives a fixed fee per person based on age and gender characteristics (Torres, 2019). Colombians with a formal job have to contribute to a compulsory health insurance program through a payroll tax. Patients out-of-pocket expenditures (premium and co-payments) are fixed for the entire system and are proportional

to their income (and even free for the poorest). These firms (insurer and purchaser) have family doctors, gatekeepers, who can prescribe and order tests, remit patients to external providers for secondary care. On top of the compulsory health insurance, individuals can buy VHI (Bardey and Buitrago, 2017).

The country economy is largely informal: nearly half of the employees have positions that do not contribute to the payroll tax (LaboUR, 2018). Hence, for those individuals the government provides a means tested subsidised regime that pays, through general taxes, their premiums. One of the central concerns of the system is that effective access is not as complete as the system promises. Unavailability of medical appointments, especially for certain specialities, and the shortage of certain medications or tests are common complaints. Given that EPS' income is given by containing costs and is not linked to clinical outcomes, incentives for quality are low (Bardey, 2015). This also implied that for a while, the quality and availability of clinical information was relatively low. As a result, there is evidence of SES gradients on effective access to health care (Lopera-Medina, 2017).

Prevalence of HBP among older individuals is around 30% among older individuals, with individuals with lower SES bearing the greatest burden of HBP (Hessel et al., 2020).

According to the World Bank indicators, Colombia had a GDP per capita of around 6.175 USD by 2010 (third of the study). The GINI coeficient for the country was around 51 in those years, second only to South Africa in this set of countries.

A.3 Ghana

Ghana has a national health insurance system with a low yearly premium. Still, as described by Drislane et al. (2014), its effectiveness is severely limited by the lack of health professionals in rural areas of the country. Because the country still suffers from high prevalence of transmissible diseases, such as malaria or tuberculosis, NCDs are not a primary focus yet. A central characteristic of Ghana's healthcare system is that medication is typically bought out-of-pocket, affecting the effective treatment of several conditions if the families are unable to buy VHI. Budget pressures are central, as the country is moving from an aid-based to contribution-based funding of its healthcare system.

Between 19% and 48% of the populations studied in the country suffer from HBP Bosu (2010). However, the central issues are awareness and control of HBP: less than one third of individuals know that they have the condition, and only one in ten have it under control. The lack of staff is a severe issue for the control of chronic conditions, and for this reason, the CBM is a promising option in the country. Interventions using local nurses and community health officers resulted in substantial improvements of HBP control rates, but low retention remains a central issue (Adler et al., 2019; Nyame et al., 2019). Moreover, there are still important challenges on how to fund effective therapies in Ghana's health system, which requires the implementation of rules that take into account cost-effectiveness and budget impact analysis (Gad et al., 2019).

This country is the second last in terms of GDP per capita (1.217 USD in 2008), and the fifth in income concentration (42.8 in 2005).

A.4 India

India provides universal access to free health insurance. As in the case of Ghana, effective access is not as good as promised and this results in often large out-of-pocket expenditures (Gupta et al., 2017). Moreover, VHI services are not widely accessible, which implies that households are paying directly for health services when needed. Until recently, health policy in India focused mainly on transmissible diseases because of pressing needs for improving safe water and sanitation coverage (Augsburg and Rodriguez-Lesmes, 2018). However, high rates of cardiovascular disease have become a paramount public health concern, and in particular controlling HBP (Gupta and Xavier, 2018).

Gupta and Xavier (2018) highlight that awareness of hypertension is an issue, though it has improved dramatically in the last decades, but still with large urban-rural differences. Moreover, Anchala et al. (2014) meta-study shows that around 33% of the urban population has hypertension, only 25% are aware of it, and controlled HBP is only around 10% of patients that are aware of having HBP. Gupta and Xavier (2018) also highlight the challenges imposed by low adherence to therapies, and as a result, most research has focused on patient behaviour rather than health system components.

India is the poorest (GDP per capita of 998.5 USD in 2008) and less income concentrated (GINI of 35.4) of the seven countries.

A.5 Mexico

Mexico has a health system segmented in different sub-systems. Employment status and ability to pay determines the affiliation to any of these sub-systems. Private insurance covers all levels of care to individuals with the ability to pay. Social security (e.g. The Mexican Social Security Institute (IMSS), the Government Workers' Social Security and Services Institute (ISSSTE)) covers workers, pensioners and their families. Seguro Popular covers population without social security. OECD (2016) identified that one of the most critical problems in Mexico's health system is these sub-systems are disconnected, each of them offers different levels of care, to different groups, at different prices, with different outcomes. As in Colombia, the informal sector is large and then the subsidised system plays a crucial role (Rodríguez et al., 2016).

As for health problems, a significant health issue in Mexico is its high obesity rates. Almost one in three adults is obese. Besides, around of 34% of obese people are currently morbidly obese, the highest degree of obesity. To address this problem, Mexico has adopted a series of policies that include interventions such as mandatory food labelling on the front of the packaging, and taxes on sugary drinks and high-calorie non-essential foods. However, the growing trend in obesity has not yet to be reversed (OECD, 2020).

In consequence, HBP is as well a relevant concern. It is one of the leading causes of death in Mexico, being the main risk factor for preventable deaths. In Mexico, around 25% of older individuals have HBP. Of these, 40% were unaware of their condition. Furthermore, from those adults who at the time of applying the survey were already aware of having high blood pressure and were also receiving pharmacological treatment (79.3%), less than half (45.6%) presented controlled blood pressure values (Campos-Nonato et al., 2018).

This country is the second richest of the sample (GDP per capita of 8003 USD in 2009), and the third more concentrated (GINI of 47.2).

A.6 Russia

The Semashko model is the heart of the Russian health care provision, with a centralised state-owned system. It provides universal access and free of charge services, but has been substantially transformed in the last decades to improve its efficiency and funding. Sheiman et al. (2018) argue that its main concerns are about equity: there are substantial differences between urban and rural areas, where availability of services and waiting-lists limit effective access.

Cardiovascular diseases are a central concern in Russia, as they have one of the highest mortality rates in the world (Leon et al., 2018). Around 48% of Russian have HBP, 20% were unaware of the condition, and 56% of those under treatment had not it under control (Rotar et al., 2019). In Russia, prevalence of smoking and heavy alcohol intake is high (Rehm and Ferreira-Borges, 2018). Effective programs targeted to improve HBP management in the rural areas have been implemented recently, as it is the case of the Yaroslavl program in polyclinics as documented by Mozheyko et al. (2017). Its aims were to retrain physicians, nurses and promote patient education about HBP. The authors recognise that the main issue could be to adopt these plans given the current governance and incentives of the local health system, as well as the lack of comprehensive and uniform electronic health records.

Russia is the richest of the sample (GDP per capita of 10.675 USD in 2010), and the second less concentrated in terms of income (GINI of 39.5)

A.7 South Africa

In South Africa, universal access to healthcare is guaranteed but under separated private and public health systems. The private system has 30% of the countries' doctors but covers less than 25% of the population (Mayosi and Benatar, 2014). Access to private providers is linked to the source of access to health insurance. VHI is expensive, making income one of the primary sources of persistent health inequalities. Additionally, the country faces a significant challenge for the high emigration rates of the health workforce (Mahlathi and Dlamini, 2015). Staff shortages are even more problematic in rural areas; there the CBM is an essential tool though the emphasis is on malnutrition and infectious diseases (Mayosi and Benatar, 2014).

Similar to other MICs, many individuals in South Africa still lack access to clean water and sanitation facilities. At the same time, the country still is confronted by high prevalence of transmissible diseases such as HIV and tuberculosis. In additions, the country faces the non-communicable diseases epidemic, which affects developed countries (Coovadia et al., 2009). HBP prevalence among individuals aged 15 and older is around 35%, with only 9% of individuals diagnosed with HBP having controlled HBP (Berry et al., 2017). This is despite the strict BP recording rules in hospitals which generate congestion due to the high volumes

of patients that they have to serve (Thorogood et al., 2019). Large studies have shown that poorer and less educated individuals are more affected by hypertension, and more likely to have it under control (Thomas et al., 2018).

The country is the fourth in terms of income in the sample (GDP per capita of 6.095 USD in 2008), but the most concentrated (GINI of 63).

Table A2: Risk Factors

	China	Colombia	Ghana	India	Mexico	Russia	South Africa
**Noncommunicable diseases**							
Age-standardized NCD mortality rate (per 100 000 population)	542.4	463	708.4	597.5	457.8	659.7	707.2
**HBP**							
Raised blood pressure (SBP>=140 OR DBP>=90) (crude estimate) (2015)	20.6 [16.0-25.6]	18.2 [13.4-23.7]	18.6 [14.3-23.5]	23.5 [19.3-28.2]	18.5 [13.9-23.8]	31.7 [25.1-38.5]	24.0 [19.1-29.4]
Raised blood pressure (SBP $\geq$ 140 OR DBP $\geq$ 90), age-standardized (%) (2015)	19.2 [14.9-24.0]	19.2 [14.2-24.7]	23.7 [18.7-29.3]	25.8 [21.3-30.7]	19.7 [14.8-25.1]	27.2 [21.2-33.6]	26.9 [21.7-32.7]
**Tobacco**							
Estimate of current tobacco use prevalence (%) (age-standardized rate) (2018)	24.7 [18.5-30.9]	7.9 [6.0-10.0]	3.7 [2.4-5.2]	27.0 [20.5-33.7]	13.9 [11.1-16.8]	28.3 [23.0-33.7]	31.4 [21.9-41.8]
Estimate of daily tobacco use prevalence (%) (age-standardized rate)	21.5 [16.5-26.8]	5.1 [4.0-6.5]	3.0 [1.8-4.6]	20.9 [14.1-28.8]	7.8 [6.1-9.7]	27.1 [22.3-33.2]	23.5 [12.9-35.0]
**Obesity**							
Prevalence of obesity among adults, BMI ≥ 30 (crude estimate) (%) (2016)	6.60%	22.10%	9.70%	3.80%	28.40%	25.70%	27%
**Physical Activity**							
Prevalence of insufficient physical activity among adults aged 18+ years (crude estimate) (%) (2016)	14.40%	43%	20.30%	33.30%	28.40%	18%	37.20%
**CVD**							
Mortality between age 30 and exact age 70 from cardiovascular diseases, cancer, diabetes or chronic respiratory diseases.	17%	15.80%	20.80%	23.30%	15.70%	25.40%	26.20%

Notes: All indicators obtained from WHO

B Additional details on the data construction

B.1 Missing patterns

Missing data corresponds mainly to the lack of biomarkers information, in particular, blood pressure. Table B1 shows that from the entire database, around one-third of the total sample do not include this information, and affect the construction of our central dependent variables: undetected and uncontrolled HBP dummies. After this, there are missing values in education level, body mass index (BMI), and the variables that constitute the wealth index; yet, such missings affect less than 2% of the dataset.

Table B2 considers that patterns behind missing values in the key independent and dependent variables. Education is the main reason behind missings in the wealth index. For China, missings in the controls are the same as in the assets index, so the base in this regression is India. For blood pressure readings (which determines the missings in columns 2 and 3), key characteristics are age, male, obese, smoking history, and living in rural areas. Conditional on those characteristics, South Africa and Russia are the less affected, and India is the most.

Table B1: Count of missings values

	Missing	Total	Percent
Male	2	24,475	0.01
Age	2	24,475	0.01
Obese BMI $\geq$ 30	358	24,475	1.46
Smoke ever	6	24,475	0.02
Education Below Primary	399	24,475	1.63
Education Primary	399	24,475	1.63
Education Above Primary	399	24,475	1.63
Lives in urban area	5	24,475	0.02
Wealth index	294	24,475	1.20
Undetected HBP	8,423	24,475	34.41
Uncontrolled HBP	8,423	24,475	34.41

Table B2: Average marginal effects on the probability of missing values

Variables	(1) Wealth index	(2) Undetected HBP	(3) Uncontrolled HBP
Male	-0.00188	0.0337***	0.0337***
	(0.00170)	(0.00681)	(0.00681)
Age	0.0000645	-0.00614***	-0.00614***
	(0.000126)	(0.000421)	(0.000421)
Obese (BMI $\geq$ 30)	0.000129	-0.111***	-0.111***
	(0.00240)	(0.00905)	(0.00905)
Smoke ever	-0.000504	0.0204***	0.0204***
	(0.00194)	(0.00690)	(0.00690)
Education: Primary	0.114***	-0.110	-0.110
	(0.00993)	(0.0949)	(0.0949)
Education: Above Primary	0.114***	-0.104	-0.104
	(0.0104)	(0.0950)	(0.0950)
Lives in urban area	0.00240	-0.0522***	-0.0522***
	(0.00222)	(0.00690)	(0.00690)
Survey year: 2009/10	0.0149***	-0.0911***	-0.0911***
	(0.00316)	(0.0105)	(0.0105)
China [Base]			
Colombia	0.0128**	-0.0207*	-0.0207*
	(0.00516)	(0.0108)	(0.0108)
Ghana	-0.0161***	0.0391***	0.0391***
	(0.00205)	(0.0127)	(0.0127)
India		0.153***	0.153***
		(0.0124)	(0.0124)
Mexico	-0.0170***	0.0342**	0.0342**
	(0.00181)	(0.0171)	(0.0171)
Russia	-0.0128***	-0.113***	-0.113***
	(0.00234)	(0.0125)	(0.0125)
South Africa	-0.0150***	-0.141***	-0.141***
	(0.00265)	(0.0123)	(0.0123)
Observations	20236	23726	23726

Clustered standard errors in parentheses
Significance: *** p<0.01, ** p<0.05, * p<0.1

B.2 Wealth Index

Table B3: Factor loadings (pattern matrix)- SABE

Variable	Description	Factor
P301_1	Type of housing. House	-0.1332
P301_2	Type of housing. Apartment.	0.2362
P301_3	Type of housing. Room in tenancy.	-0.0098
P301_4	Room in another type of structure.	-0.0879
P301_5	Indigenous housing.	-0.15
P301_6	Other type of house.	-0.1076
P302_1	Dwelling ownership status. Rent.	0.1824
P302_2	Own housing paying (Mortgage)	0.064
P302_3	Own paid housing	-0.1056
P302_4	Housing in usufruct	-0.032
P302_5	Housing in commitment	-0.008
P302_6	Possession without title (Occupant in fact)	-0.0897
P302_7	Collective property	-0.0111
P302_8	Family-owned or third-party housing	-0.0263
P303_1	Roof. Cement.	0.4317
P303_2	Roof. Eternit tile	0.2282
P303_3	Roof. Clay tile.	-0.1064
P303_4	Roof. Zinc roof tile	-0.4882
P303_5	Roof. Plastic tile.	-0.0407
P303_6	Roof. Palm or other plant material.	-0.2609
P303_7	Roof. Wood.	0.0076
P304_1	Exterior walls. Block, brick, stone, polished wood	0.7309
P304_2	Exterior walls. Tapia tread, adobe	-0.2234
P304_3	Exterior walls. Bahareque revoked	-0.2512
P304_4	Exterior walls. Bahareque without revoking	-0.2598
P304_5	Exterior walls. Crude wood, board, plank	-0.5041
P304_6	Exterior walls. Prefabricated material	-0.0374
P304_7	Exterior walls. Guadua, cane, mat, other plant material	-0.1333
P304_8	Exterior walls. Zinc, cloth, canvas, cardboard, cans, waste, plastic	-0.146
P304_9	Exterior walls. Without walls.	-0.0503
P305_1	Interior walls. Block, brick, stone, polished wood	0.7292
P305_2	Interior walls. Tapia tread, adobe	-0.2152
P305_3	Interior walls. Bahareque revoked	-0.2499
P305_4	Interior walls. Bahareque without revoking	-0.2632
P305_5	Interior walls. Crude wood, board, plank	-0.4966
P305_6	Interior walls. Prefabricated material	-0.0424
P305_7	Interior walls. Guadua, cane, mat, other plant material	-0.1327
P305_8	Interior walls. Zinc, cloth, canvas, cardboard, cans, waste, plastic	-0.152
P305_9	Interior walls. Without walls.	-0.0746
P306_1	Floors. Marble.	0.0597
P306_2	Floors. Carpet or rug from wall to wall.	0.0679
P306_3	Floors. Tile, vinyl, tablet, brick.	0.6518
P306_4	Floors. Polished and lacquered wood, park.	-0.0186
P306_5	Floors. Coarse wood, board, plank, other vegetable.	-0.2627
P306_6	Floors. Cement, gravel.	-0.3122
P306_7	Floors. Sand earth.	-0.4861
P326_1	Electric power.	-0.3677
P326_2	Natural gas connected to the public network.	-0.6255
P326_3	Aqueduct.	-0.5054
P326_4	Sewerage system.	-0.6337
status_1	Utilities fee rate Low - Estrato 1	-0.5451

Continued on next page

Variable	Description	Factor
status_2	Utilities fee rate Low-Med - Estrato 2	0.2046
status_3	Utilities fee rate Medium - Estrato 3	0.3763
status_4	Utilities fee rate High-Med - Estrato 4	0.1796
status_5	Utilities fee rate High - Estrato 5	0.105
P311	Kitchen.	-0.2456
P315_17	Cable TV	0.5587
P315_16	Internet	0.4716
P315_14	Electric shower	0.3689
P315_12	Microwave oven	0.4284
P315_11	Electric oven / gas	0.4012
P315_10	Washing machine	0.5821
P315_8	Fridge	0.5457
P315_7	Cellphone	0.2415
P315_6	Computer	0.5113
P315_4	DVD	0.4178
P315_3	Stereo system	0.4827
P315_2	Televison	0.4546
P315_1	Radio	0.1433

Table B4: Factor loadings (pattern matrix)- SAGE

Variable	Description	South_Africa	Mexico	India	Ghana	China	Russia
car	Car	0.5013	0.3389	0.2677	0.3361	-0.1568	0.2028
q0504_1	hard floor	0.6979	0.615	0.7875	0.4762	0.5049	0.3592
q0504_2	earth floor	-0.7063	-0.615	-0.7875	-0.4785	-0.5058	-0.3536
q0505_1	walls-durable material	0.7227	0.5447	0.7145	0.7394	0.5669	0.4888
q0505_2	walls-mud/aadobe	-0.5139	-0.3949	-0.6183	-0.7305	-0.5652	-0.4714
q0505_3	walls of thatch or similar materials	-0.1026	-0.322	-0.2078	-0.0535	-0.0191	-0.1133
q0505_4	plastic sheet	-0.1118	-0.0455	-0.0251	-0.0079		-0.0361
q0505_5	other wall	-0.4138	-0.2352	-0.0327	-0.002		-0.0429
q0506_1	piped private	0.726	0.0927	0.5688	0.5124	0.6107	0.7699
q0506_10	bottled water	0.0136	0.0556	0.0058	0.1091	0.1187	0.0285
q0506_11	small scale vendor	-0.1919	-0.0032	0.0013	0.0549	-0.119	-0.0118
q0506_12	tanker-truck/lorry	-0.2713	-0.0292	0.015	0.0452		-0.0906
q0506_13	surface water (river, lake, etc)			-0.0497	-0.2771		-0.0237
q0506_2	piped to yard/plot	-0.1652	-0.2962	0.2051	0.3686	-0.266	-0.4269
q0506_3	public tap/standpipe	-0.5041	-0.096	-0.0679	0.1654	-0.0663	-0.4243
q0506_4	tubewell/borehole	-0.0723	-0.1354	-0.3704	-0.4129	-0.4273	-0.1644
q0506_5	protected dug well	-0.0627	-0.2155	-0.0311	-0.0787	-0.1829	-0.1523
q0506_6	unprotected dug well	-0.0803	-0.2205	-0.1648	-0.1144	-0.1996	-0.1017
q0506_7	protected spring	-0.0151	-0.163	0.0087	0.0145	-0.067	-0.2017
q0506_8	unprotected spring	-0.1564	-0.1086	-0.0038	-0.0553	-0.1246	-0.1744
q0506_9	rainwater collection	-0.1072	0.2053	0.0082	0.0177	-0.0126	-0.0008
q0508_1	flush/pour to piped sewage system	0.7284	0.6437	0.466	0.2539	0.5928	0.7243
q0508_10	bucket	-0.1227	-0.0378	0.0226	0.1195	-0.1151	-0.0839
q0508_11	hanging toilet/latrine	-0.0407	-0.3331	-0.0277	-0.0283	-0.0405	0.0127
q0508_12	no facilities (bush, field)	-0.3518		-0.585	-0.4411		0.0261
q0508_2	flush/pour to septic tank	-0.0374	-0.3628	0.4571	0.5193	0.2318	0.0318
q0508_3	flush/pour to pit latrine	-0.11	-0.2784	0.0498	0.0745	-0.1852	-0.0675
q0508_4	flush/pour to other location	-0.0079	-0.0268	0.0747	0.023	-0.0061	-0.0954
q0508_5	flush/pour to unknown	-0.0814	-0.0631	0.0533	0.0197	-0.1215	-0.0504
q0508_6	ventilation improved pit latrine	-0.2181	-0.2441	0.0446	0.2213	-0.4798	-0.1831
q0508_7	pit with slab	-0.3053	-0.2114	0.0082	-0.1886	-0.3719	-0.3235
q0508_8	pit witout slap/open	-0.2512	-0.0991	-0.0249	-0.1604	-0.0182	-0.5143

Continued on next page

Variable	Description	South_Africa	Mexico	India	Ghana	China	Russia
q0508_9	composting toilet	-0.0462	-0.0607	-0.1805	0.019	-0.0153	-0.076
q0510_1	gas	-0.0187	0.753	0.7581	0.5065	0.7723	-0.0198
q0510_2	electricity	0.6492	-0.0494	0.0306	0.037	0.0026	0.1384
q0510_3	kerosene/paraffin	-0.4823	-0.0812	0.0721	0.0458	-0.0111	0.0049
q0510_4	coal, charcoal	-0.0547	-0.1081	0.023	0.5114	-0.0741	-0.0933
q0510_5	wood	-0.3968	-0.7451	-0.5056	-0.6969	-0.3372	-0.2641
q0510_6	Animal dung	-0.0712	-0.0332	-0.1596	-0.1601	-0.2832	-0.0881
q0510_7	shrubs, grass	-0.0405		-0.1284	0.0127	-0.0433	
q0510_8	Other			-0.1187	-0.0042	-0.4717	
q0705_1	Asset#5 Bicycle	0.2274	0.1403	-0.0004	-0.1642	0.0885	
q0708_1	Asset#8 Washing machine	0.614	0.5558		0.1028	0.6206	0.3533
q0709_1	Asset#9 Dishwasher	0.2693	0.0786	0.0861	0.1589	0.1025	0.0884
q0710_1	Asset#10 Refrigerator	0.6638	0.4825	0.6702	0.7102	0.7262	0.1988
q0711_1	Asset#11 Fixed-line telephone	0.535		0.5438	0.3643	0.3701	0.443
q0712_1	Asset#12 Mobile telephone	0.3945	0.3336	0.5728	0.5891	0.4656	0.2635
q0713_1	Asset#13 TV	0.4004			0.6984	0.3266	0.099
q0714_1	Asset#14 Computer	0.4103	0.3494	0.2879	0.3185	0.6197	0.4348
q0715_1	Asset#14 Stereo system	0.4602	0.3301	0.3919	0.2196	0.3453	
q0717_1	Asset#17 Internet access at home		0.3099			0.6007	0.4128
television	Asset #1 Television	0.6346	0.5316			0.3118	

C Additional results on the main specification

Table C1: Regressions for undetected and uncontrolled HBP conditional on being aware o hypertension by country, alternative models

Variable	OLS Systolic BP	Logit (or) Undetected	Logit (or) Uncontrolled | Diagnosis	Logit (or) Uncontrolled (Unconditional)	Multinomial Logit (rrr) Undetected	Multinomial Logit (rrr) Uncontrolled (Unconditional)
	(1)	(2)	(3)	(4)	(5)	(6)
China × Wealth index	-17.70***	-2.253***	-1.843***	1.375***	-3.713***	-1.781***
	(1.877)	(0.206)	(0.371)	(0.203)	(0.364)	(0.363)
Colombia × Wealth index	-17.43***	-1.645***	-1.109***	-0.394*	-2.150***	-1.087***
	(2.428)	(0.246)	(0.234)	(0.208)	(0.275)	(0.234)
Ghana × Wealth index	-5.693	-3.305***	-1.603**	2.480***	-4.491***	-1.520**
	(3.467)	(0.400)	(0.681)	(0.430)	(0.587)	(0.649)
India × Wealth index	-11.64***	-1.487***	-1.244**	-0.00396	-2.065***	-1.305**
	(3.872)	(0.367)	(0.547)	(0.468)	(0.455)	(0.580)
Mexico × Wealth index	-7.315	-3.669***	-0.889	2.564**	-4.380*	-0.961
	(10.37)	(1.135)	(2.114)	(1.289)	(2.236)	(2.513)
Russia × Wealth index	-18.70**	-4.037***	-3.192**	0.790	-6.632***	-3.219**
	(7.823)	(1.448)	(1.455)	(1.145)	(1.910)	(1.439)
South Africa × Wealth index	-1.414	-0.720	-0.499	0.423	-1.209	-0.610
	(4.646)	(0.463)	(0.742)	(0.510)	(0.808)	(0.896)
China						
Colombia	-20.20***	-1.740***	-4.252***	-0.0818	-4.479***	-4.168***
	(5.824)	(0.614)	(0.816)	(0.533)	(0.848)	(0.778)
Ghana	-2.195	-0.607	-0.281	0.355	-0.823	-0.192
	(6.973)	(0.738)	(1.600)	(0.767)	(1.231)	(1.278)
India	-29.74***	-1.117	-4.070***	-0.662	-3.713***	-3.838***
	(8.719)	(0.880)	(1.371)	(0.942)	(1.174)	(1.250)
Mexico	-28.43*	-1.547	-3.796	-0.631	-4.780	-4.272
	(16.17)	(1.737)	(3.023)	(1.714)	(3.237)	(3.268)
Russia	-11.36	-0.0210	0.548	1.509	0.743	0.906
	(10.28)	(1.784)	(2.250)	(1.533)	(2.582)	(2.205)
South Africa	-9.528	-1.054	-1.347	0.467	-2.123	-1.304
	(9.736)	(0.936)	(1.622)	(0.940)	(1.477)	(1.487)
Coefficients for the controls are omitted from this regression table.						
Observations	15127	15127	8767	15127	15127	

Notes: own calculations using SAGE and with sample weights. The first column presents the coefficients of an OLS regression. Second to fourth columns present the odds-ratio after logistic regressions. The fifth and sixth columns present relative-risk-ratios after a multinomial logistic regression, where the alternative is to be aware and having BP under control. The included controls are: age and being male; obesity status and smoking history; and education level (primary, and above primary), and living in an urban area. All regressions include a dummy that indicates that the individual was surveyed in year 209/10 as opposite to 2007/08, and the interaction between each control and this set of dummies. Household level clustered standard errors are presented in parentheses. Significance: * 0.1, ** 0.05, *** 0.01

Table C2: Blinder-Oaxaca decomposition, undetected HBP

	(1) **China**	(2) **Colombia**	(3) **Ghana**	(4) **India**	(5) **Mexico**	(6) **Russia**	(7) **South Africa**
overall							
group 1: Poor=0	0.449***	0.161***	0.719***	0.506***	0.412***	0.169***	0.554***
	(0.00850)	(0.00683)	(0.0129)	(0.0148)	(0.0183)	(0.00993)	(0.0143)
group 2: Poor=1	0.681***	0.263***	0.941***	0.702***	0.485***	0.229***	0.638***
	(0.0164)	(0.0168)	(0.0166)	(0.0297)	(0.0371)	(0.0224)	(0.0296)
difference	-0.233***	-0.102***	-0.221***	-0.195***	-0.0732*	-0.0597**	-0.0841**
	(0.0184)	(0.0181)	(0.0211)	(0.0332)	(0.0414)	(0.0245)	(0.0329)
explained	-0.131***	-0.0457***	-0.129***	-0.0980***	-0.0252	-0.0258***	-0.0726***
	(0.0111)	(0.0145)	(0.0148)	(0.0122)	(0.0206)	(0.00965)	(0.0155)
unexplained	-0.101***	-0.0562**	-0.0925***	-0.0974***	-0.0479	-0.0339	-0.0115
	(0.0212)	(0.0230)	(0.0236)	(0.0342)	(0.0439)	(0.0249)	(0.0355)
explained							
Age	-0.000994	0.000748	-0.000473	-0.00364	0.00106	0.00409*	0.0000188
	(0.000780)	(0.00103)	(0.000869)	(0.00268)	(0.00279)	(0.00221)	(0.000240)
Male	-0.000368	-0.00747***	-0.0244***	-0.00611	0.00991	-0.00210	0.00160
	(0.000530)	(0.00242)	(0.00681)	(0.00404)	(0.00801)	(0.00296)	(0.00308)
Smoke ever	-0.00535**	-0.000327	-0.00489	0.00122	-0.00247	-0.0000248	-0.000118
	(0.00215)	(0.000803)	(0.00458)	(0.00442)	(0.00283)	(0.000217)	(0.000637)
Obese (BMI $\geq$ 30)	-0.00403***	-0.00350***	-0.00810**	-0.00356	-0.00991*	-0.00456**	-0.00763*
	(0.00134)	(0.00132)	(0.00372)	(0.00224)	(0.00563)	(0.00226)	(0.00399)
Lives in urban area	-0.105***	-0.0310**	-0.0609***	-0.0441***	-0.000867	-0.0150**	-0.0606***
	(0.0120)	(0.0141)	(0.0115)	(0.00977)	(0.0171)	(0.00752)	(0.0137)
Education: Primary	0.000325	-0.00269	-0.00339	-0.000516	-0.00720	-0.00915	-0.00308
	(0.00151)	(0.00277)	(0.00239)	(0.00261)	(0.00507)	(0.00559)	(0.00309)
Education: Above Primary	-0.0158***	-0.00140	-0.0268***	-0.0413***	-0.0158**	0.00425	-0.00278
	(0.00606)	(0.00231)	(0.00633)	(0.00809)	(0.00803)	(0.00560)	(0.00603)
Survey year: 2009/10	-0.0000138					-0.00331	
	(0.000907)					(0.00206)	
unexplained							
Age	-0.0277	0.0757	-0.199	-0.311	0.255	0.0138	-0.296
	(0.185)	(0.152)	(0.133)	(0.303)	(0.429)	(0.261)	(0.266)
Male	-0.00217	-0.0354**	0.0347	0.0318	-0.00386	0.0589**	0.0203
	(0.0233)	(0.0169)	(0.0298)	(0.0402)	(0.0325)	(0.0232)	(0.0246)
Smoke ever	0.0000479	0.0269	0.00716	-0.0190	0.0226	-0.0566***	-0.00847
	(0.0191)	(0.0196)	(0.0153)	(0.0455)	(0.0332)	(0.0193)	(0.0250)
Obese (BMI $\geq$ 30)	0.00226	0.0151	-0.0000333	-0.00126	0.0167	0.0224	-0.0156
	(0.00377)	(0.0107)	(0.00502)	(0.00484)	(0.0251)	(0.0157)	(0.0268)
Lives in urban area	0.000856	-0.0338	-0.0102*	0.0123	-0.0105	0.0489	-0.0526*
	(0.00685)	(0.0232)	(0.00614)	(0.0113)	(0.0486)	(0.0356)	(0.0307)
Education: Primary	0.00881	0.00315	-0.00662**	0.00144	0.0229	-0.000888	-0.000350
	(0.00818)	(0.00768)	(0.00310)	(0.0146)	(0.0232)	(0.0177)	(0.0155)
Education: Above Primary	0.000633	0.000415	-0.00699	0.00201	0.0238***	-0.0431	-0.00456
	(0.00629)	(0.00336)	(0.00621)	(0.0116)	(0.00846)	(0.0654)	(0.00939)
Survey year: 2009/10	-0.0178					0.0109	
	(0.0231)					(0.0119)	
Constant	-0.0661	-0.108	0.0887	0.186	-0.375	-0.0882	0.346
	(0.187)	(0.158)	(0.146)	(0.306)	(0.442)	(0.284)	(0.283)
Observations	4476	3591	1434	1429	893	1827	1572

Household level clustered standard errors in parentheses.
Significance: *** p<0.01, ** p<0.05, * p<0.1

Table C3: Blinder-Oaxaca decomposition, uncontrolled HBP conditional on being aware o hypertension

	(1) **China**	(2) **Colombia**	(3) **Ghana**	(4) **India**	(5) **Mexico**	(6) **Russia**	(7) **South Africa**
overall							
group_1	0.762***	0.408***	0.760***	0.459***	0.717***	0.786***	0.752***
	(0.00989)	(0.00996)	(0.0232)	(0.0212)	(0.0225)	(0.0120)	(0.0187)
group_2	0.899***	0.505***	0.750***	0.521***	0.709***	0.806***	0.886***
	(0.0184)	(0.0222)	(0.125)	(0.0583)	(0.0505)	(0.0234)	(0.0307)
difference	-0.137***	-0.0971***	0.0101	-0.0619	0.00733	-0.0192	-0.134***
	(0.0209)	(0.0243)	(0.127)	(0.0621)	(0.0553)	(0.0263)	(0.0359)
explained	-0.0814***	-0.0607***	-0.0771**	-0.0279	0.00167	-0.00825	-0.0636***
	(0.0143)	(0.0194)	(0.0348)	(0.0178)	(0.0263)	(0.00968)	(0.0218)
unexplained	-0.0558**	-0.0365	0.0872	-0.0340	0.00566	-0.0109	-0.0703*
	(0.0234)	(0.0306)	(0.133)	(0.0646)	(0.0614)	(0.0273)	(0.0403)
explained							
Age	0.000821	-0.00178	-0.00504	0.000383	-0.00235	-0.00354	-0.00255
	(0.000980)	(0.00203)	(0.00767)	(0.00301)	(0.00438)	(0.00235)	(0.00308)
Male	-0.0000559	-0.00338	0.00346	-0.00100	-0.00115	-0.000569	0.0000216
	(0.000343)	(0.00206)	(0.00839)	(0.00279)	(0.00261)	(0.00118)	(0.000398)
Smoke ever	0.00241	0.00306*	-0.0132	0.00146	0.000888	-0.000302	-0.000226
	(0.00205)	(0.00170)	(0.0183)	(0.00642)	(0.00275)	(0.00101)	(0.00115)
Obese (BMI $\geq$ 30)	0.000799	0.000331	0.00249	0.00127	0.0103	0.00376	0.000144
	(0.000863)	(0.00120)	(0.00620)	(0.00301)	(0.00722)	(0.00257)	(0.00260)
Lives in urban area	-0.0814***	-0.0549***	-0.0449	-0.0204	0.00388	0.00181	-0.0230
	(0.0145)	(0.0190)	(0.0281)	(0.0145)	(0.0230)	(0.00779)	(0.0191)
Education: Primary	0.00276	-0.000475	-0.00392	-0.000530	-0.00127	0.00759	-0.00623
	(0.00172)	(0.00401)	(0.00817)	(0.00301)	(0.00452)	(0.00614)	(0.00461)
Education: Above Primary	-0.00708	-0.00353	-0.0161	-0.00907	-0.00868	-0.0104	-0.0318***
	(0.00812)	(0.00316)	(0.0140)	(0.0117)	(0.00837)	(0.00680)	(0.0102)
Survey year: 2009/10	0.000259					-0.00654**	
	(0.00472)					(0.00313)	
unexplained							
Age	0.226	-0.0830	0.900	-0.112	-0.321	-0.0139	-0.387
	(0.213)	(0.212)	(0.851)	(0.594)	(0.547)	(0.274)	(0.302)
Male	0.00618	-0.00776	0.00985	0.00458	0.0418	0.00316	-0.0327
	(0.0285)	(0.0202)	(0.0453)	(0.0747)	(0.0383)	(0.0205)	(0.0244)
Smoke ever	0.00491	0.0651**	0.139	0.0535	-0.0392	0.00566	-0.0153
	(0.0231)	(0.0263)	(0.107)	(0.0766)	(0.0384)	(0.0157)	(0.0253)
Obese (BMI $\geq$ 30)	-0.00472*	-0.0101	0.0891	-0.0152	0.0246	-0.0140	0.0316
	(0.00270)	(0.0164)	(0.0832)	(0.0107)	(0.0363)	(0.0186)	(0.0369)
Lives in urban area	-0.00211	-0.0393	-0.00215	-0.0301	0.0248	-0.0259	-0.0399
	(0.00942)	(0.0310)	(0.00851)	(0.0284)	(0.0635)	(0.0409)	(0.0393)
Education: Primary	-0.000364	-0.00961	0.00000217	0.00745	-0.0000243	-0.0501**	-0.00321
	(0.00716)	(0.0108)	(0.000462)	(0.0279)	(0.0298)	(0.0212)	(0.0214)
Education: Above Primary	0.00108	-0.00482	0.0516	-0.0606**	-0.00484	-0.146*	-0.0200**
	(0.00786)	(0.00373)	(0.0563)	(0.0281)	(0.0185)	(0.0808)	(0.00801)
Survey year: 2009/10	0.0820***					0.0101	
	(0.0277)					(0.0167)	
Constant	-0.369*	0.0530	-1.100	0.118	0.280	0.220	0.396
	(0.217)	(0.218)	(0.881)	(0.607)	(0.570)	(0.303)	(0.326)
Observations	2266	2942	358	659	513	1496	677

Household level clustered standard errors in parentheses.
Significance: *** p<0.01, ** p<0.05, * p<0.1

Table C4: Blinder-Oaxaca decomposition, undetected HBP, including health insurance status

	(1) **China**	(2) **Colombia**	(3) **Ghana**	(4) **India**	(5) **Russia**	(6) **South Africa**
overall						
group 1: Poor=0	0.449***	0.161***	0.719***	0.506***	0.169***	0.554***
	(0.00850)	(0.00683)	(0.0129)	(0.0149)	(0.00993)	(0.0143)
group 2: Poor=1	0.681***	0.263***	0.941***	0.702***	0.229***	0.638***
	(0.0164)	(0.0168)	(0.0167)	(0.0297)	(0.0224)	(0.0296)
difference	-0.233***	-0.102***	-0.221***	-0.195***	-0.0597**	-0.0841**
	(0.0185)	(0.0181)	(0.0211)	(0.0332)	(0.0245)	(0.0329)
explained	-0.130***	-0.0492***	-0.146***	-0.105***	-0.0269***	-0.0724***
	(0.0112)	(0.0146)	(0.0156)	(0.0123)	(0.00978)	(0.0156)
unexplained	-0.103***	-0.0529**	-0.0749***	-0.0903***	-0.0329	-0.0117
	(0.0212)	(0.0230)	(0.0238)	(0.0343)	(0.0248)	(0.0355)
explained						
Age	-0.000880	0.000679	-0.000298	-0.00371	0.00411*	-0.0000241
	(0.000739)	(0.000963)	(0.000718)	(0.00271)	(0.00222)	(0.000246)
Male	-0.000404	-0.00722***	-0.0245***	-0.00633	-0.00210	0.00167
	(0.000567)	(0.00237)	(0.00678)	(0.00416)	(0.00296)	(0.00321)
Smoke ever	-0.00508**	-0.000308	-0.00331	0.00127	-0.0000258	-0.000109
	(0.00213)	(0.000795)	(0.00451)	(0.00440)	(0.000225)	(0.000591)
Obese (BMI $\geq$ 30)	-0.00404***	-0.00346***	-0.00902**	-0.00339	-0.00460**	-0.00745*
	(0.00135)	(0.00132)	(0.00375)	(0.00217)	(0.00227)	(0.00391)
Lives in urban area	-0.110***	-0.0303**	-0.0606***	-0.0413***	-0.0153**	-0.0553***
	(0.0123)	(0.0141)	(0.0114)	(0.00974)	(0.00753)	(0.0137)
Education: Primary	0.000521	-0.00268	-0.00315	-0.000525	-0.00917	-0.00274
	(0.00151)	(0.00277)	(0.00234)	(0.00266)	(0.00561)	(0.00307)
Education: Above Primary	-0.0140**	-0.00128	-0.0253***	-0.0399***	0.00428	-0.0000832
	(0.00608)	(0.00235)	(0.00623)	(0.00798)	(0.00563)	(0.00605)
Voluntary Health Insurance (VIH)	0.00211	-0.00116	-0.0206	-0.00196	0.000356	0.000519
	(0.00129)	(0.00171)	(0.0156)	(0.00349)	(0.000820)	(0.00158)
No health insurance	0.00248*	-0.00347**	0.000210	-0.00921*	-0.00102	-0.00889*
	(0.00149)	(0.00172)	(0.0155)	(0.00542)	(0.00153)	(0.00470)
Survey year: 2009/10	0.000000369				-0.00334	
	(0.000789)				(0.00207)	
unexplained						
Age	-0.0147	0.0662	-0.169	-0.318	0.0494	-0.273
	(0.186)	(0.152)	(0.131)	(0.303)	(0.259)	(0.267)
Male	-0.000245	-0.0323*	0.0354	0.0343	0.0579**	0.0228
	(0.0233)	(0.0169)	(0.0297)	(0.0401)	(0.0231)	(0.0247)
Smoke ever	0.000269	0.0270	0.00623	-0.0193	-0.0573***	-0.0102
	(0.0192)	(0.0195)	(0.0153)	(0.0454)	(0.0194)	(0.0252)
Obese (BMI $\geq$ 30)	0.00214	0.0162	-0.000856	-0.00105	0.0227	-0.0154
	(0.00376)	(0.0107)	(0.00490)	(0.00483)	(0.0159)	(0.0268)
Lives in urban area	0.00242	-0.0353	-0.0106*	0.0131	0.0493	-0.0470
	(0.00699)	(0.0231)	(0.00610)	(0.0114)	(0.0357)	(0.0309)
Education: Primary	0.00811	0.00303	-0.00624**	0.00114	0.000395	-0.000989
	(0.00818)	(0.00761)	(0.00302)	(0.0146)	(0.0178)	(0.0158)
Education: Above Primary	0.000574	0.000932	-0.00667	0.00257	-0.0394	-0.00315
	(0.00627)	(0.00354)	(0.00596)	(0.0116)	(0.0654)	(0.00942)
Voluntary Health Insurance (VIH)	-0.00318	-0.00182	-0.0113	-0.0000669	0.00113	-0.00202
	(0.00391)	(0.00222)	(0.0279)	(0.000192)	(0.00107)	(0.0274)
No health insurance	-0.00104	-0.00314	0.0316	0.139*	-0.00122	0.0599
	(0.00614)	(0.00359)	(0.0660)	(0.0790)	(0.00116)	(0.218)
Survey year: 2009/10	-0.0136				0.00990	
	(0.0235)				(0.0119)	
Constant	-0.0837	-0.0937	0.0563	0.0572	-0.126	0.257
	(0.188)	(0.158)	(0.169)	(0.315)	(0.281)	(0.378)
Observations	4473	3587	1434	1429	1827	1572

Household level clustered standard errors in parentheses.
Significance: *** p<0.01, ** p<0.05, * p<0.1

Table C5: Blinder-Oaxaca decomposition, uncontrolled HBP conditional on being aware of hypertension, including health insurance status

	(1)<br>**China**	(2)<br>**Colombia**	(3)<br>**Ghana**	(4)<br>**India**	(5)<br>**Russia**	(6)<br>**South Africa**
overall						
group 1: Poor=0	0.762***	0.408***	0.760***	0.459***	0.786***	0.752***
	(0.00989)	(0.00997)	(0.0232)	(0.0213)	(0.0120)	(0.0187)
group 2: Poor=1	0.899***	0.504***	0.750***	0.521***	0.806***	0.886***
	(0.0184)	(0.0222)	(0.125)	(0.0583)	(0.0234)	(0.0309)
difference	-0.137***	-0.0961***	0.0101	-0.0619	-0.0192	-0.134***
	(0.0209)	(0.0244)	(0.127)	(0.0621)	(0.0263)	(0.0361)
explained	-0.0820***	-0.0641***	-0.0747**	-0.0240	-0.00727	-0.0638***
	(0.0146)	(0.0196)	(0.0352)	(0.0183)	(0.00982)	(0.0218)
unexplained	-0.0549**	-0.0320	0.0849	-0.0380	-0.0119	-0.0701*
	(0.0235)	(0.0307)	(0.133)	(0.0647)	(0.0271)	(0.0405)
explained						
Age	0.000735	-0.00175	-0.00519	0.000458	-0.00349	-0.00243
	(0.000934)	(0.00206)	(0.00774)	(0.00301)	(0.00234)	(0.00297)
Male	-0.0000836	-0.00321	0.00343	-0.00120	-0.000569	0.00000976
	(0.000356)	(0.00202)	(0.00832)	(0.00289)	(0.00118)	(0.000391)
Smoke ever	0.00217	0.00296*	-0.0133	0.00219	-0.000317	-0.000225
	(0.00201)	(0.00167)	(0.0184)	(0.00644)	(0.00101)	(0.00115)
Obese (BMI $\geq$ 30)	0.000830	0.000354	0.00269	0.00138	0.00383	0.000262
	(0.000875)	(0.00119)	(0.00627)	(0.00304)	(0.00261)	(0.00262)
Lives in urban area	-0.0763***	-0.0552***	-0.0448	-0.0214	0.00228	-0.0204
	(0.0146)	(0.0191)	(0.0283)	(0.0147)	(0.00776)	(0.0193)
Education: Primary	0.00267	-0.000160	-0.00392	-0.000429	0.00753	-0.00618
	(0.00169)	(0.00401)	(0.00823)	(0.00246)	(0.00615)	(0.00460)
Education: Above Primary	-0.00907	-0.00317	-0.0159	-0.00872	-0.0106	-0.0310***
	(0.00819)	(0.00319)	(0.0141)	(0.0118)	(0.00686)	(0.0101)
Voluntary Health Insurance (VIH)	-0.000853	-0.00295	0.00442	-0.0141*	-0.000399	0.000410
	(0.00180)	(0.00279)	(0.0247)	(0.00744)	(0.000905)	(0.00226)
No health insurance	-0.00230	-0.000958	-0.00225	0.0179*	0.000987	-0.00434
	(0.00192)	(0.00114)	(0.0281)	(0.0100)	(0.00164)	(0.00682)
Survey year: 2009/10	0.000195				-0.00651**	
	(0.00453)				(0.00313)	
unexplained						
Age	0.208	-0.0572	1.958	-0.107	0.0384	-0.331
	(0.212)	(0.212)	(1.414)	(0.593)	(0.273)	(0.304)
Male	0.00722	-0.00810	-0.0848	0.00636	0.00315	-0.0363
	(0.0286)	(0.0203)	(0.0730)	(0.0747)	(0.0204)	(0.0249)
Smoke ever	0.00373	0.0664**	0.205	0.0504	0.00283	-0.0129
	(0.0231)	(0.0263)	(0.129)	(0.0766)	(0.0155)	(0.0257)
Obese (BMI $\geq$ 30)	-0.00468*	-0.0103	0.0654	-0.0151	-0.0227	0.0375
	(0.00274)	(0.0165)	(0.0621)	(0.0106)	(0.0178)	(0.0373)
Lives in urban area	-0.00564	-0.0376	-0.00178	-0.0309	-0.0369	-0.0332
	(0.0102)	(0.0309)	(0.00861)	(0.0285)	(0.0403)	(0.0396)
Education: Primary	0.000154	-0.00957	0.0000101	0.00552	-0.0540**	0.00322
	(0.00722)	(0.0109)	(0.000470)	(0.0278)	(0.0211)	(0.0224)
Education: Above Primary	0.00144	-0.00504	0.0363	-0.0606**	-0.146*	-0.0202**
	(0.00787)	(0.00391)	(0.0413)	(0.0281)	(0.0810)	(0.00828)
Voluntary Health Insurance (VIH)	0.000233	0.000446	0.0174	0.000621	0.00357	0.0112
	(0.00360)	(0.00129)	(0.0708)	(0.000380)	(0.00310)	(0.0139)
No health insurance	0.00524	0.00284	-0.206	-0.185*	0.00275	0.280**
	(0.00938)	(0.00371)	(0.183)	(0.1000)	(0.00256)	(0.118)
Survey year: 2009/10	0.0845***				0.0122	
	(0.0277)				(0.0167)	
Constant	-0.355	0.0261	-1.905	0.298	0.185	0.0315
	(0.220)	(0.219)	(1.257)	(0.614)	(0.303)	(0.364)
Observations	2264	2938	358	659	1496	677

Household level clustered standard errors in parentheses.
Significance: *** p<0.01, ** p<0.05, * p<0.1

Table C6: Fairlie extension to Blinder-Oaxaca decomposition, undetected HBP

	(1) **China**	(2) **Colombia**	(3) **Ghana**	(4) **India**	(5) **Mexico**	(6) **Russia**	(7) **South Africa**
overall							
group 1: Poor=0	0.449***	0.161***	0.719***	0.506***	0.412***	0.169***	0.554***
	(0.00850)	(0.00683)	(0.0129)	(0.0148)	(0.0183)	(0.00993)	(0.0143)
group 2: Poor=1	0.681***	0.263***	0.941***	0.702***	0.485***	0.229***	0.638***
	(0.0164)	(0.0168)	(0.0166)	(0.0297)	(0.0371)	(0.0224)	(0.0296)
difference	-0.233***	-0.102***	-0.221***	-0.195***	-0.0732*	-0.0597**	-0.0841**
	(0.0184)	(0.0181)	(0.0211)	(0.0332)	(0.0414)	(0.0245)	(0.0329)
explained	-0.131***	-0.0457***	-0.129***	-0.0980***	-0.0252	-0.0258***	-0.0726***
	(0.0111)	(0.0145)	(0.0148)	(0.0122)	(0.0206)	(0.00965)	(0.0155)
unexplained	-0.101***	-0.0562**	-0.0925***	-0.0974***	-0.0479	-0.0339	-0.0115
	(0.0212)	(0.0230)	(0.0236)	(0.0342)	(0.0439)	(0.0249)	(0.0355)
explained							
Age	-0.000994	0.000748	-0.000473	-0.00364	0.00106	0.00409*	0.0000188
	(0.000780)	(0.00103)	(0.000869)	(0.00268)	(0.00279)	(0.00221)	(0.000240)
Male	-0.000368	-0.00747***	-0.0244***	-0.00611	0.00991	-0.00210	0.00160
	(0.000530)	(0.00242)	(0.00681)	(0.00404)	(0.00801)	(0.00296)	(0.00308)
Smoke ever	-0.00535**	-0.000327	-0.00489	0.00122	-0.00247	-0.0000248	-0.000118
	(0.00215)	(0.000803)	(0.00458)	(0.00442)	(0.00283)	(0.000217)	(0.000637)
Obese (BMI $\geq$ 30)	-0.00403***	-0.00350***	-0.00810**	-0.00356	-0.00991*	-0.00456**	-0.00763*
	(0.00134)	(0.00132)	(0.00372)	(0.00224)	(0.00563)	(0.00226)	(0.00399)
Lives in urban area	-0.105***	-0.0310**	-0.0609***	-0.0441***	-0.000867	-0.0150**	-0.0606***
	(0.0120)	(0.0141)	(0.0115)	(0.00977)	(0.0171)	(0.00752)	(0.0137)
Education: Primary	0.000325	-0.00269	-0.00339	-0.000516	-0.00720	-0.00915	-0.00308
	(0.00151)	(0.00277)	(0.00239)	(0.00261)	(0.00507)	(0.00559)	(0.00309)
Education: Above Primary	-0.0158***	-0.00140	-0.0268***	-0.0413***	-0.0158**	0.00425	-0.00278
	(0.00606)	(0.00231)	(0.00633)	(0.00809)	(0.00803)	(0.00560)	(0.00603)
Survey year: 2009/10	-0.0000138					-0.00331	
	(0.000907)					(0.00206)	
unexplained							
Age	-0.0277	0.0757	-0.199	-0.311	0.255	0.0138	-0.296
	(0.185)	(0.152)	(0.133)	(0.303)	(0.429)	(0.261)	(0.266)
Male	-0.00217	-0.0354**	0.0347	0.0318	-0.00386	0.0589**	0.0203
	(0.0233)	(0.0169)	(0.0298)	(0.0402)	(0.0325)	(0.0232)	(0.0246)
Smoke ever	0.0000479	0.0269	0.00716	-0.0190	0.0226	-0.0566***	-0.00847
	(0.0191)	(0.0196)	(0.0153)	(0.0455)	(0.0332)	(0.0193)	(0.0250)
Obese (BMI $\geq$ 30)	0.00226	0.0151	-0.0000333	-0.00126	0.0167	0.0224	-0.0156
	(0.00377)	(0.0107)	(0.00502)	(0.00484)	(0.0251)	(0.0157)	(0.0268)
Lives in urban area	0.000856	-0.0338	-0.0102*	0.0123	-0.0105	0.0489	-0.0526*
	(0.00685)	(0.0232)	(0.00614)	(0.0113)	(0.0486)	(0.0356)	(0.0307)
Education: Primary	0.00881	0.00315	-0.00662**	0.00144	0.0229	-0.000888	-0.000350
	(0.00818)	(0.00768)	(0.00310)	(0.0146)	(0.0232)	(0.0177)	(0.0155)
Education: Above Primary	0.000633	0.000415	-0.00699	0.00201	0.0238***	-0.0431	-0.00456
	(0.00629)	(0.00336)	(0.00621)	(0.0116)	(0.00846)	(0.0654)	(0.00939)
Survey year: 2009/10	-0.0178					0.0109	
	(0.0231)					(0.0119)	
Constant	-0.0661	-0.108	0.0887	0.186	-0.375	-0.0882	0.346
	(0.187)	(0.158)	(0.146)	(0.306)	(0.442)	(0.284)	(0.283)
Observations	4476	3591	1434	1429	893	1827	1572

Household level clustered standard errors in parentheses.
Significance: *** p<0.01, ** p<0.05, * p<0.1

Table C7: Fairlie extension to Blinder-Oaxaca decomposition, uncontrolled HBP

	(1) **China**	(2) **Colombia**	(3) **Ghana**	(4) **India**	(5) **Mexico**	(6) **Russia**	(7) **South Africa**
overall							
group 1: Poor=0	0.420***	0.342***	0.213***	0.227***	0.421***	0.653***	0.335***
	(0.00832)	(0.00881)	(0.0118)	(0.0124)	(0.0185)	(0.0126)	(0.0134)
group 2: Poor=1	0.287***	0.372***	0.0446***	0.155***	0.365***	0.621***	0.321***
	(0.0156)	(0.0184)	(0.0145)	(0.0232)	(0.0363)	(0.0252)	(0.0285)
difference	0.133***	-0.0300	0.169***	0.0712***	0.0562	0.0322	0.0147
	(0.0177)	(0.0204)	(0.0187)	(0.0263)	(0.0407)	(0.0282)	(0.0315)
explained	0.0640***	-0.0275*	0.0846***	0.0324***	0.0180	0.0137	0.0336**
	(0.0110)	(0.0166)	(0.0134)	(0.00955)	(0.0202)	(0.0112)	(0.0138)
unexplained	0.0695***	-0.00257	0.0843***	0.0388	0.0383	0.0185	-0.0189
	(0.0204)	(0.0261)	(0.0212)	(0.0275)	(0.0439)	(0.0291)	(0.0338)
explained							
Age	0.00125	-0.00138	0.0000842	0.00189	-0.00143	-0.00574*	0.000191
	(0.000879)	(0.00187)	(0.000586)	(0.00173)	(0.00374)	(0.00294)	(0.000777)
Male	0.000363	-0.00125	0.0208***	0.00245	-0.00746	0.00112	-0.00126
	(0.000524)	(0.00133)	(0.00615)	(0.00200)	(0.00614)	(0.00167)	(0.00244)
Smoke ever	0.00576***	0.00241*	-0.000351	0.000344	0.00208	0.0000393	0.000145
	(0.00216)	(0.00132)	(0.00417)	(0.00379)	(0.00273)	(0.000329)	(0.000776)
Obese (BMI $\geq$ 30)	0.00378***	0.00187	0.00680*	0.00221	0.0136**	0.00760**	0.00587*
	(0.00133)	(0.00144)	(0.00351)	(0.00184)	(0.00618)	(0.00354)	(0.00324)
Lives in urban area	0.0431***	-0.0275*	0.0386***	0.0100	0.00223	0.0129	0.0399***
	(0.0117)	(0.0162)	(0.0104)	(0.00829)	(0.0173)	(0.00879)	(0.0126)
Education: Primary	0.00151	0.000581	0.00223	0.000411	0.00428	0.0136*	-0.000301
	(0.00152)	(0.00358)	(0.00207)	(0.00208)	(0.00481)	(0.00703)	(0.00289)
Education: Above Primary	0.00818	-0.00221	0.0165***	0.0151**	0.00464	-0.0122	-0.0110*
	(0.00598)	(0.00275)	(0.00549)	(0.00620)	(0.00815)	(0.00741)	(0.00589)
Survey year: 2009/10	0.0000335	-0.00372					
	(0.00220)					(0.00266)	
unexplained							
Age	0.105	-0.101	0.131	0.131	-0.306	-0.0108	0.118
	(0.180)	(0.178)	(0.123)	(0.250)	(0.414)	(0.296)	(0.261)
Male	0.00350	0.0162	-0.0352	-0.0166	0.0233	-0.0430	-0.0263
	(0.0225)	(0.0190)	(0.0272)	(0.0314)	(0.0310)	(0.0262)	(0.0242)
Smoke ever	0.00275	0.0368	0.0192	0.0301	-0.0308	0.0487**	0.00394
	(0.0181)	(0.0227)	(0.0134)	(0.0376)	(0.0322)	(0.0209)	(0.0239)
Obese (BMI $\geq$ 30)	-0.00398	-0.0159	0.00528**	-0.00409	-0.00119	-0.0292	0.0177
	(0.00385)	(0.0133)	(0.00253)	(0.00514)	(0.0239)	(0.0189)	(0.0260)
Lives in urban area	-0.000917	-0.0175	0.0119***	-0.0154	0.0147	-0.0591	0.0275
	(0.00674)	(0.0263)	(0.00394)	(0.0111)	(0.0477)	(0.0407)	(0.0290)
Education: Primary	-0.00710	-0.00961	0.00558**	0.00482	-0.0106	-0.0426*	-0.00237
	(0.00803)	(0.00911)	(0.00280)	(0.0108)	(0.0229)	(0.0227)	(0.0155)
Education: Above Primary	-0.00177	-0.00416	0.00898***	-0.0190	-0.0169	-0.0953	-0.00507
	(0.00606)	(0.00352)	(0.00330)	(0.0117)	(0.0111)	(0.0858)	(0.00933)
Survey year: 2009/10	0.0601***					0.0000826	
	(0.0221)					(0.0152)	
Constant	-0.0878	0.0929	-0.0625	-0.0722	0.365	0.250	-0.152
	(0.182)	(0.183)	(0.137)	(0.252)	(0.424)	(0.326)	(0.279)
Observations	4476	3591	1434	1429	893	1827	1572

Household level clustered standard errors in parentheses.
Significance: *** p<0.01, ** p<0.05, * p<0.1

Figure C1: Contribution to gap in uncontrolled and undetected HBP. Differences between urban and rural areas.

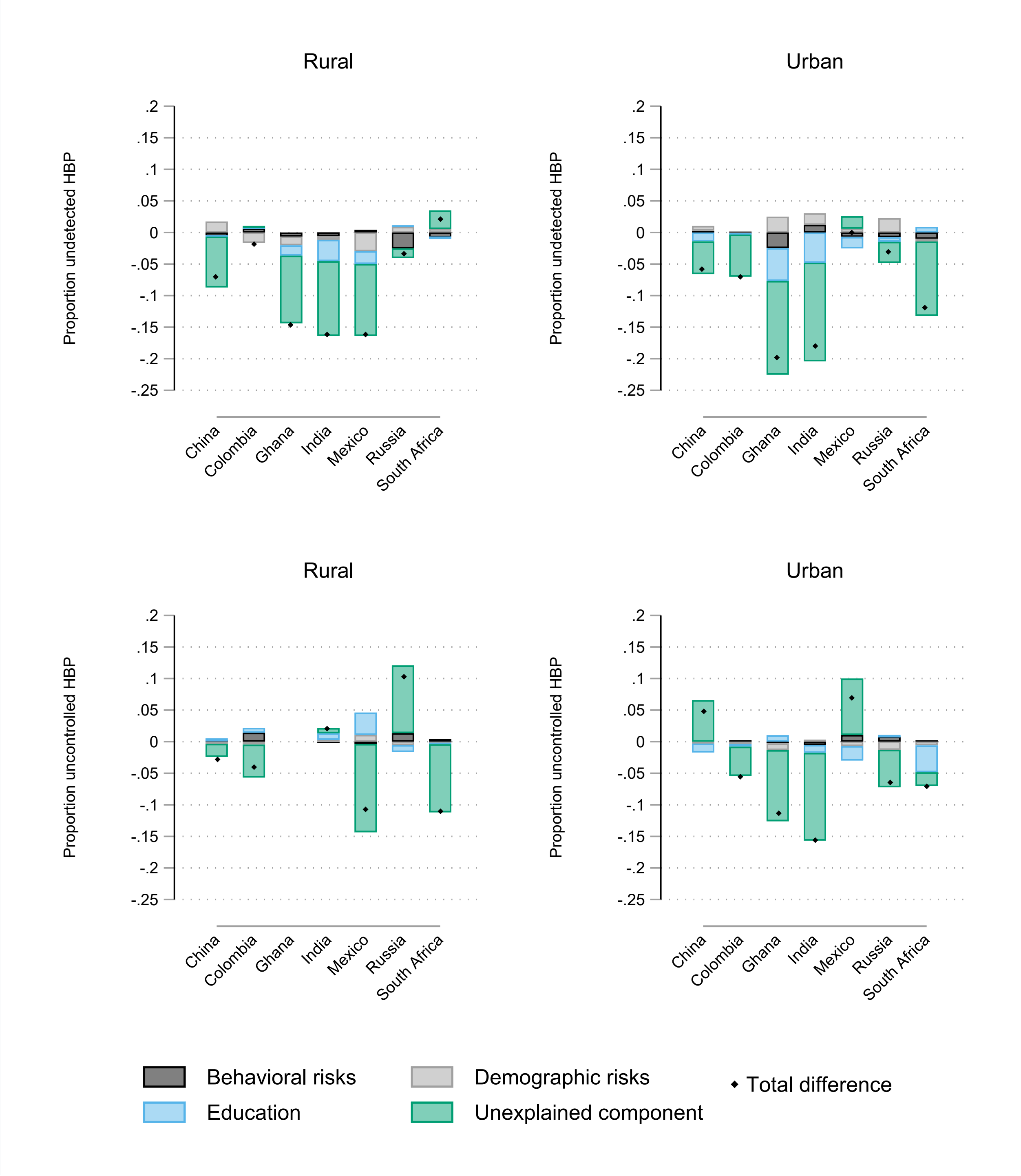

Notes: Contribution of covariate groups to the gap on undetected and uncotrolled HBP between individuals in the lowest wealth quintile agains the other quintiles. These numbers are computed with the Blinder-Oaxaca decomposition after linear probability models.

D Inclusion of individuals 50 to 59

Here we presents results including individuals 50-59 for the SAGE countries.

Table D1 presents the marginal effects of wealth by country. For China and India the relationships are stepper, for Ghana, Russia and South Africa are less pronounced. The significance of the specification with health insurance for uncontrolled HBP changes for Ghana and India, but it is due to the precision of the estimate rather than the size of the coefficients. Yet, the qualitative results are the same, as well as the dependence on the inclusion of covariates.

As for the BO decomposition, tables D2 and D3 present the results extending the age range. For Colombia there are no changes, as SABE does not include individuals 50-59. In general, there is almost no difference with the main results presented in the main text in terms of the magnitudes, the proportion explained, and the role of each variable. Though, for South Africa the gap in undetected HBP is almost half of the version that does not include the under 60. However, differences on education seems to be the main reason behind.

Table D1: Average marginal effects of wealth on undetected and uncontrolled HBP, by country. Including individuals 50 to 59.

Country	$\frac{\partial \text{Undetected}}{\partial \text{Wealth}}$				$\frac{\partial \text{Uncontrolled}}{\partial \text{Wealth}} \mid$ Diagnosis =1			
	(1)	(2)	(3)	(4)	(5)	(6)	(7)	(8)
China	-0.525 ***	-0.503 ***	-0.450 ***	-0.222 ***	-0.323 ***	-0.315 ***	-0.283 ***	0.046
	(0.029)	(0.029)	(0.033)	(0.045)	(0.046)	(0.045)	(0.051)	(0.067)
Colombia	-0.274 ***	-0.273 ***	-0.283 ***	-0.272 ***	-0.250 ***	-0.260 ***	-0.258 ***	-0.161 **
	(0.039)	(0.039)	(0.045)	(0.058)	(0.050)	(0.050)	(0.054)	(0.069)
Ghana	-0.749 ***	-0.702 ***	-0.600 ***	-0.529 ***	-0.328 ***	-0.318 ***	-0.351 ***	-0.259 *
	(0.049)	(0.052)	(0.061)	(0.077)	(0.113)	(0.110)	(0.111)	(0.138)
India	-0.417 ***	-0.405 ***	-0.272 ***	-0.198 **	-0.161 *	-0.135	-0.212 *	-0.223
	(0.056)	(0.055)	(0.067)	(0.085)	(0.095)	(0.094)	(0.111)	(0.145)
Mexico	-0.207	-0.160	-0.090		-0.180	-0.336	-0.254	
	(0.221)	(0.232)	(0.235)		(0.313)	(0.289)	(0.348)	
Russia	-0.656 ***	-0.615 ***	-0.586 ***	-0.519 ***	-0.406 ***	-0.461 ***	-0.435 ***	-0.431 ***
	(0.197)	(0.179)	(0.205)	(0.171)	(0.119)	(0.120)	(0.122)	(0.156)
South Africa	-0.189 ***	-0.171 ***	-0.174 **	-0.103	-0.152	-0.151	-0.091	-0.010
	(0.065)	(0.063)	(0.069)	(0.077)	(0.094)	(0.098)	(0.103)	(0.102)
Observations	21827	21827	21827	20740	11434	11434	11434	10819
Age and gender	X	X	X	X	X	X	X	X
Obesity and smoking		X	X	X		X	X	X
Education, urban			X	X			X	X
Health Insurance				X				X

Notes: own calculations using SABE and SAGE studies with sample weights. Average marginal effects after logistic regressions are presented in the table. In each estimated equation, each region/country was multiplied by the wealth index in order to obtain a specific gradient. Controls differ according to columns: (i) age and being male; (ii) obesity status and smoking history; and (iii) education level (primary, and above primary), living in an urban area, having voluntary health insurance, and not having health insurance at all. All regressions include country dummies and the interaction between each control and this set of dummies. Standard errors are presented in parenthesis. Significance: * 0.1, ** 0.05, *** 0.01

Table D2: Blinder-Oaxaca decomposition, undetected HBP, including 50-59 for SAGE countries

	(1) **China**	(2) **Colombia**	(3) **Ghana**	(4) **India**	(5) **Mexico**	(6) **Russia**	(7) **South Africa**
overall							
group 1: Poor=0	0.502***	0.161***	0.740***	0.514***	0.427***	0.205***	0.588***
	(0.00682)	(0.00683)	(0.0100)	(0.0113)	(0.0168)	(0.00892)	(0.0110)
group 2: Poor=1	0.703***	0.263***	0.931***	0.732***	0.468***	0.258***	0.676***
	(0.0126)	(0.0168)	(0.0139)	(0.0225)	(0.0338)	(0.0198)	(0.0214)
difference	-0.201***	-0.102***	-0.191***	-0.218***	-0.0411	-0.0527**	-0.0880***
	(0.0143)	(0.0181)	(0.0171)	(0.0252)	(0.0377)	(0.0217)	(0.0241)
explained	-0.120***	-0.0457***	-0.135***	-0.0832***	-0.0235	-0.0145	-0.0409***
	(0.00829)	(0.0145)	(0.0121)	(0.00987)	(0.0188)	(0.00925)	(0.0108)
unexplained	-0.0812***	-0.0562**	-0.0559***	-0.135***	-0.0176	-0.0382*	-0.0471*
	(0.0159)	(0.0230)	(0.0192)	(0.0264)	(0.0402)	(0.0221)	(0.0259)
explained							
Age	0.00122	0.000748	0.00147	-0.00262	0.00142	0.0115***	-0.00156
	(0.00167)	(0.00103)	(0.00236)	(0.00219)	(0.00308)	(0.00353)	(0.00205)
Male	-0.000600	-0.00747***	-0.0172***	-0.00338	0.00527	-0.00348	0.00411**
	(0.000475)	(0.00242)	(0.00428)	(0.00372)	(0.00796)	(0.00258)	(0.00198)
Smoke ever	-0.00282**	-0.000327	-0.00413	-0.000670	-0.00269	-0.000359	0.0000195
	(0.00126)	(0.000803)	(0.00319)	(0.00347)	(0.00263)	(0.000621)	(0.000458)
Obese (BMI $\geq$ 30)	-0.00504***	-0.00350***	-0.0137***	-0.00275*	-0.0109*	-0.00793***	-0.00958***
	(0.00121)	(0.00132)	(0.00375)	(0.00154)	(0.00578)	(0.00249)	(0.00308)
Lives in urban area	-0.103***	-0.0310**	-0.0622***	-0.0263***	0.00668	-0.00571	-0.0365***
	(0.00868)	(0.0141)	(0.00952)	(0.00759)	(0.0153)	(0.00673)	(0.00922)
Education: Primary	0.000146	-0.00269	-0.00486**	-0.00265	-0.00690	-0.00785*	-0.00143
	(0.000510)	(0.00277)	(0.00211)	(0.00187)	(0.00460)	(0.00460)	(0.00147)
Education: Above Primary	-0.0102**	-0.00140	-0.0341***	-0.0448***	-0.0163**	0.00260	0.00407
	(0.00487)	(0.00231)	(0.00594)	(0.00668)	(0.00750)	(0.00465)	(0.00527)
Survey year: 2009/10	-0.00000999					-0.00321*	
	(0.000895)					(0.00164)	
unexplained							
Age	-0.196*	0.0757	-0.329***	-0.0566	-0.258	-0.0430	-0.0538
	(0.103)	(0.152)	(0.0882)	(0.169)	(0.316)	(0.160)	(0.148)
Male	-0.0156	-0.0354**	0.0346	0.0490*	0.00320	0.0325	0.00619
	(0.0183)	(0.0169)	(0.0234)	(0.0279)	(0.0325)	(0.0206)	(0.0175)
Smoke ever	-0.00541	0.0269	-0.00334	-0.0286	0.00210	-0.0447**	-0.000866
	(0.0145)	(0.0196)	(0.0117)	(0.0339)	(0.0324)	(0.0184)	(0.0186)
Obese (BMI $\geq$ 30)	0.00709**	0.0151	-0.00341	-0.00158	0.00900	0.0220	-0.00204
	(0.00323)	(0.0107)	(0.00343)	(0.00418)	(0.0218)	(0.0141)	(0.0192)
Lives in urban area	0.000625	-0.0338	-0.00622	0.00506	0.00271	0.0455	-0.0360
	(0.00462)	(0.0232)	(0.00534)	(0.00677)	(0.0446)	(0.0290)	(0.0245)
Education: Primary	0.0105	0.00315	-0.00470	0.00221	0.0194	-0.00122	0.00482
	(0.00741)	(0.00768)	(0.00487)	(0.00998)	(0.0208)	(0.0128)	(0.0123)
Education: Above Primary	-0.00193	0.000415	-0.00702	-0.00670	0.0161*	-0.0539	-0.00614
	(0.00651)	(0.00336)	(0.00639)	(0.00940)	(0.00955)	(0.0681)	(0.00821)
Survey year: 2009/10	-0.0102					-0.00409	
	(0.0173)					(0.0115)	
Constant	0.130	-0.108	0.264***	-0.0976	0.188	0.00857	0.0407
	(0.104)	(0.158)	(0.0957)	(0.173)	(0.331)	(0.193)	(0.160)
Observations	7096	3591	2333	2438	1072	2637	2740

Household level clustered standard errors in parentheses.
Significance: *** p<0.01, ** p<0.05, * p<0.1

Table D3: Blinder-Oaxaca decomposition, uncontrolled HBP conditional on being aware of hypertension, including 50-59 for SAGE countries

	(1) **China**	(2) **Colombia**	(3) **Ghana**	(4) **India**	(5) **Mexico**	(6) **Russia**	(7) **South Africa**
overall							
group 1: Poor=0	0.761***	0.408***	0.750***	0.452***	0.711***	0.760***	0.757***
	(0.00827)	(0.00996)	(0.0192)	(0.0161)	(0.0208)	(0.0107)	(0.0148)
group 2: Poor=1	0.880***	0.505***	0.826***	0.463***	0.676***	0.801***	0.820***
	(0.0163)	(0.0222)	(0.0791)	(0.0475)	(0.0463)	(0.0206)	(0.0297)
difference	-0.119***	-0.0971***	-0.0761	-0.0110	0.0349	-0.0406*	-0.0626*
	(0.0183)	(0.0243)	(0.0814)	(0.0501)	(0.0507)	(0.0232)	(0.0332)
explained	-0.0884***	-0.0607***	-0.0612**	-0.0193	-0.000819	-0.000489	-0.0426***
	(0.0112)	(0.0194)	(0.0302)	(0.0143)	(0.0238)	(0.00925)	(0.0159)
unexplained	-0.0309	-0.0365	-0.0149	0.00835	0.0357	-0.0401*	-0.0200
	(0.0200)	(0.0306)	(0.0856)	(0.0519)	(0.0558)	(0.0242)	(0.0361)
explained							
Age	0.000319	-0.00178	-0.0000401	0.0000994	0.00118	-0.00899***	-0.000502
	(0.000595)	(0.00203)	(0.00471)	(0.00132)	(0.00426)	(0.00328)	(0.00111)
Male	-0.000194	-0.00338	0.00313	-0.000675	-0.000903	-0.000811	0.000750
	(0.000721)	(0.00206)	(0.00614)	(0.00157)	(0.00200)	(0.00129)	(0.00136)
Smoke ever	0.000665	0.00306*	-0.00168	-0.00318	0.00164	-0.000515	0.000378
	(0.000806)	(0.00170)	(0.0110)	(0.00470)	(0.00276)	(0.000857)	(0.00154)
Obese (BMI $\geq$ 30)	0.000400	0.000331	0.00513	-0.000679	0.0108	0.00723**	0.000356
	(0.000563)	(0.00120)	(0.00874)	(0.00244)	(0.00735)	(0.00299)	(0.00240)
Lives in urban area	-0.0783***	-0.0549***	-0.0654***	-0.0169	-0.00199	0.00929	-0.0107
	(0.0120)	(0.0190)	(0.0245)	(0.0121)	(0.0201)	(0.00728)	(0.0134)
Education: Primary	0.000134	-0.000475	0.00211	0.000485	-0.00305	0.00539	-0.00430
	(0.000805)	(0.00401)	(0.00383)	(0.00267)	(0.00423)	(0.00521)	(0.00330)
Education: Above Primary	-0.00945	-0.00353	-0.00450	0.00154	-0.00851	-0.00732	-0.0286***
	(0.00719)	(0.00316)	(0.0123)	(0.0101)	(0.00832)	(0.00588)	(0.00843)
Survey year: 2009/10	-0.00203					-0.00475**	
	(0.00282)					(0.00234)	
unexplained							
Age	-0.146	-0.0830	-0.334	-0.278	-0.449	0.192	-0.312
	(0.142)	(0.212)	(0.519)	(0.371)	(0.396)	(0.173)	(0.250)
Male	0.00539	-0.00776	0.0712	0.0791	0.0128	-0.0134	-0.0378*
	(0.0227)	(0.0202)	(0.0694)	(0.0507)	(0.0355)	(0.0184)	(0.0204)
Smoke ever	-0.00562	0.0651**	0.0979	0.00677	-0.00837	0.00828	0.0209
	(0.0178)	(0.0263)	(0.0612)	(0.0583)	(0.0377)	(0.0164)	(0.0251)
Obese (BMI $\geq$ 30)	0.000467	-0.0101	0.0482	-0.00711	0.0346	0.000942	0.0536*
	(0.00457)	(0.0164)	(0.0447)	(0.00821)	(0.0322)	(0.0170)	(0.0310)
Lives in urban area	-0.00307	-0.0393	-0.00704	-0.0256	0.0404	-0.0221	-0.0373
	(0.00733)	(0.0310)	(0.00809)	(0.0175)	(0.0593)	(0.0330)	(0.0372)
Education: Primary	-0.00388	-0.00961	-0.0366	-0.0151	0.0198	-0.0470***	0.0000512
	(0.00855)	(0.0108)	(0.0339)	(0.0206)	(0.0284)	(0.0166)	(0.0207)
Education: Above Primary	0.00180	-0.00482	-0.00114	-0.0345	-0.00103	-0.218**	-0.0164**
	(0.00901)	(0.00373)	(0.0313)	(0.0224)	(0.0171)	(0.0862)	(0.00829)
Survey year: 2009/10	0.0400					-0.00918	
	(0.0253)					(0.0143)	
Constant	0.0796	0.0530	0.147	0.283	0.387	0.0676	0.308
	(0.150)	(0.218)	(0.543)	(0.370)	(0.416)	(0.218)	(0.270)
Observations	3251	2942	543	1097	606	2069	1082

Household level clustered standard errors in parentheses.
Significance: *** p<0.01, ** p<0.05, * p<0.1

E Alternative wealth index

Our sample selection presented a particular definition of the wealth index based on assets. In this section we consider an alternative version of the index that includes the total income of the household, which is available in the harmonised SAGE dataset.

Table E1 considers the new index for the marginal effects analysis. First, for Colombia there is almost no difference as SABE does not include a monetary income variable. Second, the magnitude of the coefficients appear to be larger, however, this is mostly by construction as the new index has a smaller standard deviation (0.12) than the original index (0.26). Thus, the -1.093 coefficient for China (column 1) says that a change of 1 SD on the wealth index will reduce the probability of detection in 13 pp.; in the original exercise (coefficient of -0.585), 1 SD of the the wealth index would reduce the same probability in 15 pp. Therefore, differences are not large, and qualitative results hold.

Tables E2 and E3 present the BO decomposition using the alternative asset variable. There is almost no difference at all. The reason for this is that income might change the sorting of individuals on the top of the distribution, but has little impact on the lowest quantiles. Therefore, the classification of 'non-poor' barely changes. While not explored here in details, this is likely to happen with financial assets, which are likely to be null or too small for the lowest part of the distribution.

Table E1: Average marginal effects of wealth on undetected and uncontrolled HBP conditional on being aware of hypertension, by region and country. Alternative wealth index.

Country	$\frac{\partial \text{Undetected}}{\partial \text{Wealth}}$				$\frac{\partial \text{Uncontrolled}}{\partial \text{Wealth}} \mid$ Diagnosis=1			
	(1)	(2)	(3)	(4)	(5)	(6)	(7)	(8)
China	-1.093 ***	-1.050 ***	-0.929 ***	-0.553 ***	-0.609 ***	-0.607 ***	-0.574 ***	0.044
	(0.068)	(0.069)	(0.080)	(0.106)	(0.098)	(0.100)	(0.112)	(0.150)
Colombia	-0.242 ***	-0.243 ***	-0.253 ***	-0.254 ***	-0.259 ***	-0.269 ***	-0.268 ***	-0.167 **
	(0.033)	(0.035)	(0.040)	(0.055)	(0.052)	(0.053)	(0.056)	(0.072)
Ghana	-1.108 ***	-1.070 ***	-1.023 ***	-0.765 ***	-0.427 ***	-0.394 ***	-0.379 ***	-0.382
	(0.096)	(0.098)	(0.104)	(0.160)	(0.153)	(0.142)	(0.140)	(0.248)
India	-1.025 ***	-1.027 ***	-0.725 ***	-0.586 ***	-0.528 **	-0.589 **	-0.641 **	-0.590 *
	(0.144)	(0.151)	(0.175)	(0.204)	(0.255)	(0.267)	(0.276)	(0.328)
Mexico	-0.945 ***	-0.985 ***	-1.067 ***		-0.598	-0.721	-0.326	
	(0.281)	(0.285)	(0.271)		(0.559)	(0.567)	(0.576)	
Russia	-0.667 ***	-0.631 ***	-0.541 ***	-0.409 **	-0.355 *	-0.426 **	-0.391 **	-0.451 **
	(0.207)	(0.209)	(0.193)	(0.200)	(0.183)	(0.193)	(0.179)	(0.216)
South Africa	-0.474 **	-0.443 **	-0.362 *	-0.127	-0.244	-0.248	-0.178	-0.002
	(0.197)	(0.197)	(0.219)	(0.237)	(0.236)	(0.242)	(0.244)	(0.220)
Observations	15109	15109	15109	14205	8761	8761	8761	8241
Age and gender	X	X	X	X	X	X	X	X
Obesity and smoking		X	X	X		X	X	X
Education, urban			X	X			X	X
Health Insurance				X				X

Notes: own calculations using SABE and SAGE studies with sample weights. Average marginal effects after logistic regressions are presented in the table. In each estimated equation, each region/country was multiplied by the wealth index in order to obtain a specific gradient. Controls differ according to columns: (i) age and being male; (ii) obesity status and smoking history; and (iii) education level (primary, and above primary), living in an urban area, having voluntary health insurance, and not having health insurance at all. All regressions include country dummies and the interaction between each control and this set of dummies. Standard errors are presented in parenthesis. Significance: * 0.1, ** 0.05, *** 0.01

Table E2: Blinder-Oaxaca decomposition, undetected HBP, alternative wealth index

	(1)<br>**China**	(2)<br>**Colombia**	(3)<br>**Ghana**	(4)<br>**India**	(5)<br>**Mexico**	(6)<br>**Russia**	(7)<br>**South Africa**
overall							
group_1	0.449***	0.161***	0.720***	0.499***	0.413***	0.169***	0.555***
	(0.00850)	(0.00683)	(0.0129)	(0.0149)	(0.0183)	(0.00993)	(0.0143)
group_2	0.681***	0.263***	0.931***	0.722***	0.479***	0.229***	0.633***
	(0.0164)	(0.0168)	(0.0177)	(0.0287)	(0.0370)	(0.0224)	(0.0297)
difference	-0.233***	-0.102***	-0.212***	-0.223***	-0.0658	-0.0597**	-0.0779**
	(0.0184)	(0.0181)	(0.0219)	(0.0323)	(0.0412)	(0.0245)	(0.0330)
explained	-0.131***	-0.0457***	-0.129***	-0.0964***	-0.0237	-0.0258***	-0.0717***
	(0.0111)	(0.0145)	(0.0148)	(0.0123)	(0.0191)	(0.00965)	(0.0153)
unexplained	-0.101***	-0.0562**	-0.0823***	-0.127***	-0.0422	-0.0339	-0.00627
	(0.0212)	(0.0230)	(0.0242)	(0.0335)	(0.0433)	(0.0249)	(0.0355)
explained							
Age	-0.000994	0.000748	-0.000766	-0.00377	0.00329	0.00409*	0.00000506
	(0.000780)	(0.00103)	(0.00117)	(0.00263)	(0.00326)	(0.00221)	(0.000171)
Male	-0.000368	-0.00747***	-0.0198***	-0.00209	0.0115	-0.00210	0.00227
	(0.000530)	(0.00242)	(0.00600)	(0.00366)	(0.00813)	(0.00296)	(0.00313)
Smoke ever	-0.00535**	-0.000327	-0.00474	0.00139	-0.00273	-0.0000248	0.000146
	(0.00215)	(0.000803)	(0.00448)	(0.00397)	(0.00304)	(0.000217)	(0.000630)
Obese (BMI $\geq$ 30)	-0.00403***	-0.00350***	-0.00930**	-0.00298	-0.00952*	-0.00456**	-0.00715*
	(0.00134)	(0.00132)	(0.00411)	(0.00218)	(0.00543)	(0.00226)	(0.00393)
Lives in urban area	-0.105***	-0.0310**	-0.0635***	-0.0446***	-0.00211	-0.0150**	-0.0600***
	(0.0120)	(0.0141)	(0.0118)	(0.0102)	(0.0148)	(0.00752)	(0.0134)
Education: Primary	0.000325	-0.00269	-0.00382	-0.000949	-0.00888	-0.00915	-0.00340
	(0.00151)	(0.00277)	(0.00262)	(0.00242)	(0.00614)	(0.00559)	(0.00326)
Education: Above Primary	-0.0158***	-0.00140	-0.0273***	-0.0434***	-0.0151**	0.00425	-0.00348
	(0.00606)	(0.00231)	(0.00635)	(0.00836)	(0.00772)	(0.00560)	(0.00621)
Survey year: 2009/10	-0.0000138					-0.00331	
	(0.000907)					(0.00206)	
unexplained							
Age	-0.0277	0.0757	-0.250*	-0.526*	0.305	0.0138	-0.216
	(0.185)	(0.152)	(0.141)	(0.296)	(0.428)	(0.261)	(0.270)
Male	-0.00217	-0.0354**	0.0381	0.0388	0.0200	0.0589**	0.0216
	(0.0233)	(0.0169)	(0.0301)	(0.0372)	(0.0331)	(0.0232)	(0.0245)
Smoke ever	0.0000479	0.0269	0.00986	-0.00337	0.0275	-0.0566***	-0.00488
	(0.0191)	(0.0196)	(0.0171)	(0.0424)	(0.0319)	(0.0193)	(0.0246)
Obese (BMI $\geq$ 30)	0.00226	0.0151	0.00161	0.00188	0.0233	0.0224	-0.0202
	(0.00377)	(0.0107)	(0.00467)	(0.00535)	(0.0256)	(0.0157)	(0.0270)
Lives in urban area	0.000856	-0.0338	-0.00829	0.0151	-0.00453	0.0489	-0.0520*
	(0.00685)	(0.0232)	(0.00559)	(0.0102)	(0.0500)	(0.0356)	(0.0313)
Education: Primary	0.00881	0.00315	-0.00693**	0.00105	0.00442	-0.000888	0.000767
	(0.00818)	(0.00768)	(0.00300)	(0.0141)	(0.0210)	(0.0177)	(0.0155)
Education: Above Primary	0.000633	0.000415	-0.00811	0.00335	0.0242***	-0.0431	-0.00363
	(0.00629)	(0.00336)	(0.00625)	(0.0104)	(0.00856)	(0.0654)	(0.00937)
Survey year: 2009/10	-0.0178					0.0109	
	(0.0231)					(0.0119)	
Constant	-0.0661	-0.108	0.141	0.342	-0.442	-0.0882	0.268
	(0.187)	(0.158)	(0.155)	(0.298)	(0.442)	(0.284)	(0.288)
Observations	4476	3591	1432	1421	893	1827	1564

Household level clustered standard errors in parentheses.
Significance: *** p<0.01, ** p<0.05, * p<0.1

Table E3: Blinder-Oaxaca decomposition, uncontrolled HBP conditional on being aware of hypertension, alternative wealth index

	(1)<br>**China**	(2)<br>**Colombia**	(3)<br>**Ghana**	(4)<br>**India**	(5)<br>**Mexico**	(6)<br>**Russia**	(7)<br>**South Africa**
overall							
group 1: poor=0	0.762***	0.408***	0.759***	0.455***	0.718***	0.786***	0.750***
	(0.00989)	(0.00996)	(0.0233)	(0.0212)	(0.0225)	(0.0120)	(0.0188)
group 2: poor=1	0.899***	0.505***	0.786***	0.559***	0.701***	0.806***	0.896***
	(0.0184)	(0.0222)	(0.110)	(0.0589)	(0.0507)	(0.0234)	(0.0291)
difference	-0.137***	-0.0971***	-0.0270	-0.104*	0.0172	-0.0192	-0.147***
	(0.0209)	(0.0243)	(0.112)	(0.0626)	(0.0554)	(0.0263)	(0.0347)
explained	-0.0814***	-0.0607***	-0.0833**	-0.0254	-0.00355	-0.00825	-0.0635***
	(0.0143)	(0.0194)	(0.0379)	(0.0190)	(0.0246)	(0.00968)	(0.0212)
unexplained	-0.0558**	-0.0365	0.0563	-0.0784	0.0207	-0.0109	-0.0832**
	(0.0234)	(0.0306)	(0.118)	(0.0653)	(0.0605)	(0.0273)	(0.0389)
explained							
Age	0.000821	-0.00178	-0.00600	0.000781	-0.00466	-0.00354	-0.00208
	(0.000980)	(0.00203)	(0.00877)	(0.00504)	(0.00494)	(0.00235)	(0.00303)
Male	-0.0000559	-0.00338	0.00414	-0.000633	-0.000608	-0.000569	0.0000495
	(0.000343)	(0.00206)	(0.00834)	(0.00192)	(0.00171)	(0.00118)	(0.000400)
Smoke ever	0.00241	0.00306*	-0.0184	0.00201	0.000535	-0.000302	-0.000314
	(0.00205)	(0.00170)	(0.0187)	(0.00690)	(0.00180)	(0.00101)	(0.00132)
Obese (BMI $\geq$ 30)	0.000799	0.000331	0.00281	0.000609	0.00953	0.00376	-0.0000303
	(0.000863)	(0.00120)	(0.00687)	(0.00171)	(0.00684)	(0.00257)	(0.00283)
Lives in urban area	-0.0814***	-0.0549***	-0.0451	-0.0194	0.00215	0.00181	-0.0231
	(0.0145)	(0.0190)	(0.0289)	(0.0149)	(0.0200)	(0.00779)	(0.0184)
Education: Primary	0.00276	-0.000475	-0.00383	-0.0000729	-0.00232	0.00759	-0.00629
	(0.00172)	(0.00401)	(0.00823)	(0.00340)	(0.00733)	(0.00614)	(0.00470)
Education: Above Primary	-0.00708	-0.00353	-0.0169	-0.00872	-0.00817	-0.0104	-0.0317***
	(0.00812)	(0.00316)	(0.0144)	(0.0122)	(0.00775)	(0.00680)	(0.0103)
Survey year: 2009/10	0.000259					-0.00654**	
	(0.00472)					(0.00313)	
unexplained							
Age	0.226	-0.0830	1.274	-0.546	-0.498	-0.0139	-0.526*
	(0.213)	(0.212)	(0.976)	(0.627)	(0.537)	(0.274)	(0.283)
Male	0.00618	-0.00776	0.0642	-0.0294	0.0111	0.00316	-0.0266
	(0.0285)	(0.0202)	(0.0700)	(0.0715)	(0.0367)	(0.0205)	(0.0234)
Smoke ever	0.00491	0.0651**	0.103	0.0161	-0.0325	0.00566	-0.0331
	(0.0231)	(0.0263)	(0.0673)	(0.0782)	(0.0371)	(0.0157)	(0.0215)
Obese (BMI $\geq$ 30)	-0.00472*	-0.0101	0.0791	-0.0208*	0.00278	-0.0140	0.0149
	(0.00270)	(0.0164)	(0.0740)	(0.0123)	(0.0360)	(0.0186)	(0.0340)
Lives in urban area	-0.00211	-0.0393	-0.00119	-0.0385	0.0222	-0.0259	-0.0363
	(0.00942)	(0.0310)	(0.00784)	(0.0266)	(0.0662)	(0.0409)	(0.0384)
Education: Primary	-0.000364	-0.00961	0.0000894	0.0145	0.0287	-0.0501**	-0.000374
	(0.00716)	(0.0108)	(0.000483)	(0.0275)	(0.0276)	(0.0212)	(0.0211)
Education: Above Primary	0.00108	-0.00482	0.0553	-0.0463*	0.00707	-0.146*	-0.0193**
	(0.00786)	(0.00373)	(0.0536)	(0.0262)	(0.0201)	(0.0808)	(0.00785)
Survey year: 2009/10	0.0820***					0.0101	
	(0.0277)					(0.0167)	
Constant	-0.369*	0.0530	-1.518	0.572	0.479	0.220	0.543*
	(0.217)	(0.218)	(1.027)	(0.634)	(0.561)	(0.303)	(0.307)
Observations	2266	2942	358	657	513	1496	673

Household level clustered standard errors in parentheses.
Significance: *** p<0.01, ** p<0.05, * p<0.1

Table F1: Average marginal effects of wealth on uncontrolled HBP by country, unconditional on the diagnosis of hypertension

Country	$\frac{\partial \text{Uncontrolled}}{\partial \text{Wealth}}$ (1)	(2)	(3)	(4)
China	0.354 ***	0.338 ***	0.307 ***	0.275 ***
	(0.037)	(0.038)	(0.043)	(0.056)
Colombia	-0.085 *	-0.093 **	-0.089 *	-0.021
	(0.044)	(0.044)	(0.047)	(0.060)
Ghana	0.548 ***	0.536 ***	0.495 ***	0.395 ***
	(0.077)	(0.080)	(0.090)	(0.108)
India	0.097	0.085	-0.001	-0.015
	(0.081)	(0.086)	(0.085)	(0.092)
Mexico	0.293	0.286	0.385 ***	
	(0.201)	(0.203)	(0.140)	
Russia	0.332	0.205	0.173	-0.028
	(0.259)	(0.259)	(0.257)	(0.228)
South Africa	0.119	0.105	0.092	0.025
	(0.100)	(0.099)	(0.110)	(0.118)
Observations	15127	15127	15127	14223
Age and gender	X	X	X	X
Obesity and smoking		X	X	X
Education, urban			X	X
Health Insurance				X

Notes: own calculations using SABE and SAGE studies with individual sample weights. Average marginal effects after logistic regressions are presented in the table. In each estimated equation, each region/country was multiplied by the wealth index in order to obtain a specific gradient. Controls differ according to columns: (i) age and being male; (ii) obesity status and smoking history; and (iii) education level (primary, and above primary), living in an urban area, having voluntary health insurance, and not having health insurance at all. All regressions include country dummies, a dummy that indicates that the individual was surveyed in year 2009/10 as opposite to 2007/08, and the interaction between each control and this set of dummies. Household level clustered standard errors are presented in parentheses. Significance: * 0.1, ** 0.05, *** 0.01

F Uncontrolled HBP unconditional results

Table F2 considers the BO analysis for the unconditional version of the variable. That is, it includes as a reference group those individuals who have high records for their BP but who are unaware of the condition. Here the gap goes in the other direction for China, disappear for Colombia and appears for Ghana. The reason is that the size of the differences on non detection dominates the results. For example, the share of the population at risk of China's uncontrolled HBP for the poor is smaller than for the non-poor; however this is masked by the fact that 70% of the poor have undiagnosed HBP while this figure is only 50% for the non-poor. When we condition for HBP being detected, then we realise that 90% of those poor diagnosed with HBP have their BP uncontrolled while this figure is 76% for the non-poor. A similar reasoning is behind the results in table F1, but for the average marginal effects of the wealth index on the probabilities to have uncontrolled HBP.

Table F2: Blinder-Oaxaca decomposition, uncontrolled HBP, not conditional on diagnosis of hypertension

	(1)	(2)	(3)	(4)	(5)	(6)	(7)
	**China**	**Colombia**	**Ghana**	**India**	**Mexico**	**Russia**	**South Africa**
overall							
group 1: Poor=0	0.420***	0.342***	0.213***	0.227***	0.421***	0.653***	0.335***
	(0.00832)	(0.00881)	(0.0118)	(0.0124)	(0.0185)	(0.0126)	(0.0134)
group 2: Poor=1	0.287***	0.372***	0.0446***	0.155***	0.365***	0.621***	0.321***
	(0.0156)	(0.0184)	(0.0145)	(0.0232)	(0.0363)	(0.0252)	(0.0285)
difference	0.133***	-0.0300	0.169***	0.0712***	0.0562	0.0322	0.0147
	(0.0177)	(0.0204)	(0.0187)	(0.0263)	(0.0407)	(0.0282)	(0.0315)
explained	0.0640***	-0.0275*	0.0846***	0.0324***	0.0180	0.0137	0.0336**
	(0.0110)	(0.0166)	(0.0134)	(0.00955)	(0.0202)	(0.0112)	(0.0138)
unexplained	0.0695***	-0.00257	0.0843***	0.0388	0.0383	0.0185	-0.0189
	(0.0204)	(0.0261)	(0.0212)	(0.0275)	(0.0439)	(0.0291)	(0.0338)
explained							
Age	0.00125	-0.00138	0.0000842	0.00189	-0.00143	-0.00574*	0.000191
	(0.000879)	(0.00187)	(0.000586)	(0.00173)	(0.00374)	(0.00294)	(0.000777)
Male	0.000363	-0.00125	0.0208***	0.00245	-0.00746	0.00112	-0.00126
	(0.000524)	(0.00133)	(0.00615)	(0.00200)	(0.00614)	(0.00167)	(0.00244)
Smoke ever	0.00576***	0.00241*	-0.000351	0.000344	0.00208	0.0000393	0.000145
	(0.00216)	(0.00132)	(0.00417)	(0.00379)	(0.00273)	(0.000329)	(0.000776)
Obese (BMI $\geq$ 30)	0.00378***	0.00187	0.00680*	0.00221	0.0136**	0.00760**	0.00587*
	(0.00133)	(0.00144)	(0.00351)	(0.00184)	(0.00618)	(0.00354)	(0.00324)
Lives in urban area	0.0431***	-0.0275*	0.0386***	0.0100	0.00223	0.0129	0.0399***
	(0.0117)	(0.0162)	(0.0104)	(0.00829)	(0.0173)	(0.00879)	(0.0126)
Education: Primary	0.00151	0.000581	0.00223	0.000411	0.00428	0.0136*	-0.000301
	(0.00152)	(0.00358)	(0.00207)	(0.00208)	(0.00481)	(0.00703)	(0.00289)
Education: Above Primary	0.00818	-0.00221	0.0165***	0.0151**	0.00464	-0.0122	-0.0110*
	(0.00598)	(0.00275)	(0.00549)	(0.00620)	(0.00815)	(0.00741)	(0.00589)
Survey year: 2009/10	0.0000335					-0.00372	
	(0.00220)					(0.00266)	
unexplained							
Age	0.105	-0.101	0.131	0.131	-0.306	-0.0108	0.118
	(0.180)	(0.178)	(0.123)	(0.250)	(0.414)	(0.296)	(0.261)
Male	0.00350	0.0162	-0.0352	-0.0166	0.0233	-0.0430	-0.0263
	(0.0225)	(0.0190)	(0.0272)	(0.0314)	(0.0310)	(0.0262)	(0.0242)
Smoke ever	0.00275	0.0368	0.0192	0.0301	-0.0308	0.0487**	0.00394
	(0.0181)	(0.0227)	(0.0134)	(0.0376)	(0.0322)	(0.0209)	(0.0239)
Obese (BMI $\geq$ 30)	-0.00398	-0.0159	0.00528**	-0.00409	-0.00119	-0.0292	0.0177
	(0.00385)	(0.0133)	(0.00253)	(0.00514)	(0.0239)	(0.0189)	(0.0260)
Lives in urban area	-0.000917	-0.0175	0.0119***	-0.0154	0.0147	-0.0591	0.0275
	(0.00674)	(0.0263)	(0.00394)	(0.0111)	(0.0477)	(0.0407)	(0.0290)
Education: Primary	-0.00710	-0.00961	0.00558**	0.00482	-0.0106	-0.0426*	-0.00237
	(0.00803)	(0.00911)	(0.00280)	(0.0108)	(0.0229)	(0.0227)	(0.0155)
Education: Above Primary	-0.00177	-0.00416	0.00898***	-0.0190	-0.0169	-0.0953	-0.00507
	(0.00606)	(0.00352)	(0.00330)	(0.0117)	(0.0111)	(0.0858)	(0.00933)
Survey year: 2009/10	0.0601***					0.0000826	
	(0.0221)					(0.0152)	
Constant	-0.0878	0.0929	-0.0625	-0.0722	0.365	0.250	-0.152
	(0.182)	(0.183)	(0.137)	(0.252)	(0.424)	(0.326)	(0.279)
Observations	4476	3591	1434	1429	893	1827	1572

Household level clustered standard errors in parentheses.
Significance: *** p<0.01, ** p<0.05, * p<0.1

G Alternative definitions of the “poor” group

Tables G1 and G2 consider alternatives to the ”lowest quintile” definition of the gap. The undetected HBP gap is stable across definitions, and also the proportions explained (mostly by living in an urban area and education level above primary) and not explained. The size of the gap in uncontrolled HBP is higher when tighter definitions of the poor groups. The size of the poor group under the lowest decile definition for Ghana is too small to perform the BO decomposition.

Table G1: Blinder-Oaxaca decomposition, undetected HBP, multiple definition of groups

	(1) **China**	(2) **Colombia**	(3) **Ghana**	(4) **India**	(5) **Mexico**	(6) **Russia**	(7) **South Africa**
**A. Lowest half**							
overall							
difference	-0.221***	-0.0912***	-0.205***	-0.215***	-0.0798**	-0.0315*	-0.148***
	(0.0150)	(0.0128)	(0.0216)	(0.0262)	(0.0327)	(0.0183)	(0.0255)
explained	-0.121***	-0.0203**	-0.106***	-0.108***	-0.00605	-0.0130*	-0.0453**
	(0.0157)	(0.00867)	(0.0150)	(0.0164)	(0.0132)	(0.00748)	(0.0187)
unexplained	-0.100***	-0.0709***	-0.0986***	-0.107***	-0.0737**	-0.0186	-0.103***
	(0.0220)	(0.0147)	(0.0248)	(0.0304)	(0.0336)	(0.0187)	(0.0311)
explained							
Lives in urban area	-0.0921***	-0.0169**	-0.0534***	-0.0510***	-0.000284	-0.0106**	-0.0431***
	(0.0157)	(0.00766)	(0.0126)	(0.0148)	(0.00789)	(0.00502)	(0.0150)
Education: Above Primary	-0.0205***	0.000312	-0.0273***	-0.0478***	-0.0119*	0.00393	0.00554
	(0.00765)	(0.00285)	(0.00672)	(0.00918)	(0.00712)	(0.00551)	(0.00916)
Observations	4476	3591	1434	1429	893	1827	1572
**B. Lowest tercile**							
overall							
difference	-0.225***	-0.0924***	-0.196***	-0.174***	-0.0687**	-0.0676***	-0.123***
	(0.0157)	(0.0145)	(0.0212)	(0.0286)	(0.0346)	(0.0202)	(0.0274)
explained	-0.122***	-0.0316**	-0.115***	-0.104***	-0.0193	-0.0161**	-0.0664***
	(0.0131)	(0.0125)	(0.0147)	(0.0138)	(0.0155)	(0.00812)	(0.0179)
unexplained	-0.103***	-0.0608***	-0.0808***	-0.0694**	-0.0494	-0.0515**	-0.0563*
	(0.0203)	(0.0187)	(0.0240)	(0.0307)	(0.0356)	(0.0202)	(0.0320)
explained							
Lives in urban area	-0.0925***	-0.0217*	-0.0602***	-0.0514***	-0.00110	-0.0105*	-0.0574***
	(0.0137)	(0.0118)	(0.0127)	(0.0117)	(0.0107)	(0.00626)	(0.0157)
Education: Above Primary	-0.0197***	-0.000827	-0.0266***	-0.0488***	-0.0147*	0.00450	-0.000738
	(0.00654)	(0.00245)	(0.00627)	(0.00890)	(0.00807)	(0.00559)	(0.00712)
Observations	4476	3591	1434	1429	893	1827	1572
**C. Lowest quartile**							
overall							
difference	-0.230***	-0.0985***	-0.192***	-0.186***	-0.0644*	-0.0665***	-0.104***
	(0.0171)	(0.0165)	(0.0221)	(0.0308)	(0.0378)	(0.0224)	(0.0301)
explained	-0.123***	-0.0399***	-0.127***	-0.0977***	-0.0236	-0.0232***	-0.0698***
	(0.0117)	(0.0145)	(0.0151)	(0.0123)	(0.0178)	(0.00880)	(0.0168)
unexplained	-0.107***	-0.0586***	-0.0651***	-0.0887***	-0.0408	-0.0433*	-0.0339
	(0.0205)	(0.0217)	(0.0248)	(0.0321)	(0.0392)	(0.0228)	(0.0338)
explained							
Lives in urban area	-0.0942***	-0.0266*	-0.0665***	-0.0451***	-0.00148	-0.0127*	-0.0619***
	(0.0123)	(0.0140)	(0.0128)	(0.0101)	(0.0144)	(0.00707)	(0.0151)
Education: Above Primary	-0.0190***	-0.00122	-0.0260***	-0.0424***	-0.0150*	0.00354	-0.00207
	(0.00632)	(0.00231)	(0.00611)	(0.00815)	(0.00769)	(0.00493)	(0.00611)
Observations	4476	3591	1434	1429	893	1827	1572
**D. Lowest quintile (default)**							
overall							
difference	-0.233***	-0.102***	-0.221***	-0.195***	-0.0732*	-0.0597**	-0.0841**
	(0.0184)	(0.0181)	(0.0211)	(0.0332)	(0.0414)	(0.0245)	(0.0329)
explained	-0.126***	-0.0457***	-0.129***	-0.0980***	-0.0252	-0.0222**	-0.0726***
	(0.0110)	(0.0145)	(0.0148)	(0.0122)	(0.0206)	(0.00941)	(0.0155)
unexplained	-0.107***	-0.0562**	-0.0925***	-0.0974***	-0.0479	-0.0375	-0.0115
	(0.0212)	(0.0230)	(0.0236)	(0.0342)	(0.0439)	(0.0249)	(0.0355)
explained							
Lives in urban area	-0.0961***	-0.0310**	-0.0609***	-0.0441***	-0.000867	-0.0143*	-0.0606***
	(0.0116)	(0.0141)	(0.0115)	(0.00977)	(0.0171)	(0.00749)	(0.0137)
Education: Above Primary	-0.0187***	-0.00140	-0.0268***	-0.0413***	-0.0158**	0.00397	-0.00278
	(0.00600)	(0.00231)	(0.00633)	(0.00809)	(0.00803)	(0.00560)	(0.00603)
Observations	4476	3591	1434	1429	893	1827	1572
**E. Lowest decile**							
overall							
difference	-0.236***	-0.113***	-0.240***	-0.155***	-0.139**	-0.0174	-0.0707
	(0.0238)	(0.0256)	(0.0206)	(0.0429)	(0.0561)	(0.0308)	(0.0438)
explained	-0.127***	-0.0596***	-0.135***	-0.101***	-0.0363	-0.0250**	-0.0733***
	(0.00982)	(0.0143)	(0.0158)	(0.0128)	(0.0247)	(0.0116)	(0.0161)
unexplained	-0.108***	-0.0533*	-0.105***	-0.0538	-0.103*	0.00753	0.00254
	(0.0255)	(0.0290)	(0.0239)	(0.0424)	(0.0590)	(0.0307)	(0.0458)
explained							
Lives in urban area	-0.0980***	-0.0432***	-0.0601***	-0.0437***	0.00188	-0.0195**	-0.0598***
	(0.0103)	(0.0139)	(0.0111)	(0.00935)	(0.0211)	(0.00831)	(0.0135)
Education: Above Primary	-0.0180***	-0.00143	-0.0238***	-0.0427***	-0.0150**	0.00493	-0.00286
	(0.00536)	(0.00178)	(0.00660)	(0.00852)	(0.00766)	(0.00823)	(0.00550)
Observations	4476	3591	1434	1429	893	1827	1572

Household level clustered standard errors in parentheses.
Significance: *** p<0.01, ** p<0.05, * p<0.1

Table G2: Blinder-Oaxaca decomposition, uncontrolled HBP conditional on being aware of hypertension, multiple definition of groups

	(1) **China**	(2) **Colombia**	(3) **Ghana**	(4) **India**	(5) **Mexico**	(6) **Russia**	(7) **South Africa**
**A. Lowest half**							
overall							
difference	-0.102***	-0.102***	-0.0552	-0.0851**	-0.00123	-0.0808***	-0.0965***
	(0.0177)	(0.0182)	(0.0516)	(0.0419)	(0.0411)	(0.0211)	(0.0324)
explained	-0.129***	-0.0244**	-0.0488	-0.0262	-0.0152	0.00295	-0.0710***
	(0.0178)	(0.0113)	(0.0330)	(0.0248)	(0.0164)	(0.00727)	(0.0241)
unexplained	0.0273	-0.0778***	-0.00638	-0.0588	0.0140	-0.0837***	-0.0256
	(0.0262)	(0.0211)	(0.0591)	(0.0481)	(0.0441)	(0.0218)	(0.0390)
explained							
Lives in urban area	-0.134***	-0.0241**	-0.0373	-0.0209	0.00162	0.00595	-0.0257
	(0.0173)	(0.0100)	(0.0287)	(0.0232)	(0.0110)	(0.00532)	(0.0188)
Education: Above Primary	0.00197	-0.00178	-0.0162	-0.00878	-0.00695	-0.00825	-0.0385***
	(0.00982)	(0.00367)	(0.0138)	(0.0136)	(0.00664)	(0.00641)	(0.0121)
Observations	2266	2942	358	659	513	1496	677
**B. Lowest tercile**							
overall							
difference	-0.134***	-0.0904***	-0.137**	-0.0986**	0.00292	-0.0435*	-0.0524
	(0.0180)	(0.0200)	(0.0559)	(0.0477)	(0.0442)	(0.0223)	(0.0367)
explained	-0.111***	-0.0429***	-0.0452	-0.0252	-0.00645	0.000919	-0.0829***
	(0.0146)	(0.0165)	(0.0349)	(0.0222)	(0.0172)	(0.00795)	(0.0231)
unexplained	-0.0233	-0.0476*	-0.0921	-0.0734	0.00936	-0.0445*	0.0305
	(0.0217)	(0.0256)	(0.0648)	(0.0521)	(0.0468)	(0.0231)	(0.0421)
explained							
Lives in urban area	-0.120***	-0.0394**	-0.0278	-0.0203	0.00211	0.00499	-0.0417**
	(0.0153)	(0.0157)	(0.0307)	(0.0187)	(0.0125)	(0.00666)	(0.0203)
Education: Above Primary	0.00475	-0.00304	-0.0139	-0.00925	-0.00780	-0.00947	-0.0347***
	(0.00902)	(0.00321)	(0.0133)	(0.0147)	(0.00745)	(0.00615)	(0.0104)
Observations	2266	2942	358	659	513	1496	677
**C. Lowest quartile**							
overall							
difference	-0.134***	-0.0962***	-0.135**	-0.119**	-0.00181	-0.0300	-0.106***
	(0.0195)	(0.0223)	(0.0672)	(0.0532)	(0.0492)	(0.0243)	(0.0369)
explained	-0.109***	-0.0545***	-0.0445	-0.0231	-0.000938	0.00173	-0.0632***
	(0.0135)	(0.0193)	(0.0346)	(0.0190)	(0.0223)	(0.00869)	(0.0224)
unexplained	-0.0245	-0.0417	-0.0901	-0.0955*	-0.000876	-0.0317	-0.0423
	(0.0219)	(0.0290)	(0.0761)	(0.0562)	(0.0540)	(0.0257)	(0.0429)
explained							
Lives in urban area	-0.120***	-0.0490***	-0.0336	-0.0182	0.00386	0.00441	-0.0264
	(0.0143)	(0.0188)	(0.0308)	(0.0153)	(0.0188)	(0.00753)	(0.0202)
Education: Above Primary	0.00430	-0.00331	-0.0126	-0.00760	-0.00774	-0.00940	-0.0294***
	(0.00853)	(0.00311)	(0.0119)	(0.0127)	(0.00763)	(0.00594)	(0.00957)
Observations	2266	2942	358	659	513	1496	677
**D. Lowest quintile (default)**							
overall							
difference	-0.137***	-0.0971***	0.0101	-0.0619	0.00733	-0.0192	-0.134***
	(0.0209)	(0.0243)	(0.127)	(0.0621)	(0.0553)	(0.0263)	(0.0359)
explained	-0.106***	-0.0607***	-0.0771**	-0.0279	0.00167	-0.00153	-0.0636***
	(0.0131)	(0.0194)	(0.0348)	(0.0178)	(0.0263)	(0.00918)	(0.0218)
unexplained	-0.0312	-0.0365	0.0872	-0.0340	0.00566	-0.0176	-0.0703*
	(0.0228)	(0.0306)	(0.133)	(0.0646)	(0.0614)	(0.0273)	(0.0403)
explained							
Lives in urban area	-0.119***	-0.0549***	-0.0449	-0.0204	0.00388	0.00335	-0.0230
	(0.0140)	(0.0190)	(0.0281)	(0.0145)	(0.0230)	(0.00779)	(0.0191)
Education: Above Primary	0.00410	-0.00353	-0.0161	-0.00907	-0.00868	-0.0111	-0.0318***
	(0.00815)	(0.00316)	(0.0140)	(0.0117)	(0.00837)	(0.00687)	(0.0102)
Observations	2266	2942	358	659	513	1496	677
**E. Lowest decile**							
overall							
difference	-0.143***	-0.105***		-0.113	0.0417	0.0170	-0.149***
	(0.0279)	(0.0337)		(0.0782)	(0.0831)	(0.0362)	(0.0420)
explained	-0.108***	-0.0749***		-0.0224	-0.00241	-0.00936	-0.0652***
	(0.0126)	(0.0196)		(0.0187)	(0.0320)	(0.0107)	(0.0227)
unexplained	-0.0348	-0.0301		-0.0904	0.0441	0.0263	-0.0835*
	(0.0290)	(0.0383)		(0.0810)	(0.0902)	(0.0371)	(0.0468)
explained							
Lives in urban area	-0.121***	-0.0681***		-0.0180	0.00202	0.000969	-0.0272
	(0.0136)	(0.0194)		(0.0132)	(0.0277)	(0.00788)	(0.0197)
Education: Above Primary	0.00344	-0.00312		-0.00791	-0.00863	-0.0189*	-0.0272***
	(0.00760)	(0.00261)		(0.0108)	(0.00828)	(0.0108)	(0.00989)
Observations	2266	2942	358	659	513	1496	677

Household level clustered standard errors in parentheses.
Significance: *** p<0.01, ** p<0.05, * p<0.1

H Separate regressions per country and marginal effects of wealth

In this exercise, instead of considering a single logit regression per set of controls (one per column of table 2 in the main text), we use country specific regressions. The table H1 shows that results are similar to the main ones.

Table H1: Average marginal effects of wealth on undetected and uncontrolled HBP by country , single regressions per country

Country	$\frac{\partial \text{Undetected}}{\partial \text{Wealth}}$				$\frac{\partial \text{Uncontrolled}}{\partial \text{Wealth}}$			
	(1)	(2)	(3)	(4)	(5)	(6)	(7)	(8)
China	-0.599 ***	-0.578 ***	-0.509 ***	-0.312 ***	0.374 ***	0.358 ***	0.330 ***	0.270 ***
	(0.037)	(0.038)	(0.043)	(0.058)	(0.039)	(0.039)	(0.045)	(0.059)
Colombia	-0.236 ***	-0.230 ***	-0.234 ***	-0.218 ***	-0.083 *	-0.092 **	-0.088 *	-0.022
	(0.032)	(0.032)	(0.034)	(0.047)	(0.043)	(0.043)	(0.046)	(0.060)
Ghana	-0.610 ***	-0.597 ***	-0.555 ***	-0.508 ***	0.405 ***	0.396 ***	0.368 ***	0.341 ***
	(0.051)	(0.053)	(0.061)	(0.072)	(0.052)	(0.053)	(0.062)	(0.072)
India	-0.462 ***	-0.468 ***	-0.333 ***	-0.270 ***	0.086	0.074	-0.001	-0.014
	(0.065)	(0.069)	(0.078)	(0.093)	(0.067)	(0.072)	(0.072)	(0.083)
Mexico	-0.738 ***	-0.770 ***	-0.847 ***		0.365	0.361	0.586 *	
	(0.258)	(0.270)	(0.248)		(0.306)	(0.308)	(0.300)	
Russia	-0.757 ***	-0.675 ***	-0.649 ***	-0.436 **	0.395	0.267	0.246	0.039
	(0.255)	(0.239)	(0.246)	(0.214)	(0.271)	(0.256)	(0.258)	(0.249)
South Africa	-0.220 **	-0.211 **	-0.170	-0.050	0.117	0.108	0.095	0.026
	(0.097)	(0.099)	(0.109)	(0.117)	(0.100)	(0.102)	(0.114)	(0.121)
Age and gender	X	X	X	X	X	X	X	X
Obesity and smoking		X	X	X		X	X	X
Education, urban			X	X			X	X
Health Insurance				X				X

Notes: own calculations using SABE and SAGE studies with individual sample weights. Average marginal effects after logistic regressions are presented in the table. In each estimated equation (one per country and per set of controls), the wealth index marginal effect is presented. Controls differ according to columns: (i) age and being male; (ii) obesity status and smoking history; and (iii) education level (primary, and above primary), living in an urban area, having voluntary health insurance, and not having health insurance at all. All regressions include country dummies, a dummy that indicates that the individual was surveyed in year 209/10 as opposite to 2007/08. Household level clustered standard errors are presented in parentheses. Significance: * 0.1, ** 0.05, *** 0.01

References

Adler, A. J., Laar, A., Prieto-Merino, D., Der, R. M., Mangortey, D., Dirks, R., Lamptey, P., and Perel, P. (2019). Can a nurse-led community-based model of hypertension care improve hypertension control in ghana? results from the comhip cohort study. *BMJ open*, 9(4):e026799.

Alshamsan, R., Lee, J. T., Rana, S., Areabi, H., and Millett, C. (2017). Comparative health system performance in six middle-income countries: cross-sectional analysis using world health organization study of global ageing and health. *Journal of the Royal Society of Medicine*, 110(9):365–375.

Anchala, R., Kannuri, N. K., Pant, H., Khan, H., Franco, O. H., Di Angelantonio, E., and Prabhakaran, D. (2014). Hypertension in india: a systematic review and meta-analysis of prevalence, awareness, and control of hypertension. *Journal of hypertension*, 32(6):1170.

Augsburg, B. and Rodriguez-Lesmes, P. A. (2018). Sanitation and child health in india. *World Development*, 107:22–39.

Bardey, D. (2015). Pago por desempeño en el sistema de salud colombiano. *Revista Monitor Estratégico*, 7:4–7.

Bardey, D. and Buitrago, G. (2017). Supplemental health insurance in the colombian managed care system: Adverse or advantageous selection? *Journal of health economics*, 56:317–329.

Basu, S. and Millett, C. (2013). Social epidemiology of hypertension in middle-income countries: determinants of prevalence, diagnosis, treatment, and control in the who sage study. *Hypertension*, 62(1):18–26.

Berry, K. M., Parker, W.-a., Mchiza, Z. J., Sewpaul, R., Labadarios, D., Rosen, S., and Stokes, A. (2017). Quantifying unmet need for hypertension care in south africa through a care cascade: evidence from the sanhanes, 2011-2012. *BMJ global health*, 2(3):e000348.

Bosu, W. K. (2010). Epidemic of hypertension in ghana: a systematic review. *BMC public health*, 10(1):418.

Campos-Nonato, I., Hernández-Barrera, L., Pedroza-Tobías, A., Medina, C., and Barquera, S. (2018). Hipertensión arterial en adultos mexicanos: prevalencia, diagnóstico y tipo de tratamiento. ensanut mc 2016. *salud pública de méxico*, 60:233–243.

Coovadia, H., Jewkes, R., Barron, P., Sanders, D., and McIntyre, D. (2009). The health and health system of south africa: historical roots of current public health challenges. *The Lancet*, 374(9692):817–834.

Drislane, F. W., Akpalu, A., and Wegdam, H. H. (2014). The medical system in ghana. *The Yale journal of biology and medicine*, 87(3):321.

Fang, H. and Commonwealth Fund (2017). The chinese health care system. international health care system profiles. URL: https://international.commonwealthfund.org/countries/china/. Accessed: 2020-01-28.

Feng, X. L., Pang, M., and Beard, J. (2013). Health system strengthening and hypertension awareness, treatment and control: data from the china health and retirement longitudinal study. *Bulletin of the World Health Organization*, 92:29–41.

Gad, M., Lord, J., Chalkidou, K., Asare, B., Lutterodt, M. G., and Ruiz, F. (2019). Supporting the development of evidence-informed policy options: An economic evaluation of hypertension management in ghana. *Value in Health*.

Guo, J., Zhu, Y.-C., Chen, Y.-P., Hu, Y., Tang, X.-W., and Zhang, B. (2015). The dynamics of hypertension prevalence, awareness, treatment, control and associated factors in chinese adults: results from chns 1991–2011. *Journal of hypertension*, 33(8):1688–1696.

Gupta, I., Bhatia, M., and Commonwealth Fund (2017). The indian health care system. international health care system profiles. URL: https://international.commonwealthfund.org/countries/india/. Accessed: 2020-01-28.

Gupta, R. and Xavier, D. (2018). Hypertension: The most important non communicable disease risk factor in india. *Indian heart journal*, 70(4):565–572.

Hessel, P., Rodríguez-Lesmes, P., and Torres, D. (2020). Socio-economic inequalities in high blood pressure and additional risk factors for cardiovascular disease among older individuals in colombia: Results from a nationally representative study. *PLoS One*, 15(6):e0234326.

Huang, K., Song, Y. T., He, Y. H., and Feng, X. L. (2016). Health system strengthening and hypertension management in china. *Global health research and policy*, 1(1):13.

LaboUR, O. L. (2018). Perfil actual de la informalidad laboral en colombia: estructura y retos. *Universidad del Rosario, Observatorio Laboral Laboral de la Universidad del Rosario, Informe*, (6).

Leon, D., Malyutina, S., Kudryavtsev, A., Voevoda, M., Bobrova, N., Shiekh, S., Kholmatova, K., McKee, M., Kontsevaya, A., Diez Benavente, E., et al. (2018). Dissecting hypertension in russia: identifying aetiological and behavioural factors associated with treatment and control. *European Journal of Public Health*, 28(suppl_4):cky213–876.

Li, G., Cai, A.-P., Mo, Y.-J., Chen, J.-Y., Wei, R.-B., Huang, Y.-Q., Tang, S.-T., Zhou, Y.-L., and Feng, Y.-Q. (2015). Effects of guideline-based hypertension management in rural areas of guangdong province. *Chinese medical journal*, 128(6):799.

Lopera-Medina, M. M. (2017). Utilización de servicios de salud por enfermedades catastróficas o de alto costo en antioquia. *Revista Gerencia y Políticas de Salud*, 16(32):120–137.

Mahlathi, P. and Dlamini, J. (2015). Minimum data sets for human resources for health and the surgical workforce in south africa's health system: a rapid analysis of stock and migration. *Pretoria, South Africa: African Institute of Health and Leadership Development and WHO.*

Mayosi, B. M. and Benatar, S. R. (2014). Health and health care in south africa—20 years after mandela. *New England Journal of Medicine*, 371(14):1344–1353.

Mozheyko, M., Eregin, S., Danilenko, N., Vigdorchik, A., Tobe, S. W., Campbell, N., McLean, D., Baskakova, Z., Klimovskaia, I., Ramanathan, K., et al. (2017). Hypertension in russia: changes observed after 4 years of a comprehensive health system improvement program in the yaroslavl region. *The Journal of Clinical Hypertension*, 19(2):198–204.

Niu, H., Tian, M., Ma, A., Wang, C., and Zhang, L. (2014). Differences and determinants in access to essential public health services in china: a case study with hypertension people and under-sixes as target population. *Chinese medical journal*, 127(9):1626–1632.

Nyame, S., Iwelunmor, J., Ogedegbe, G., Adjei, K. G. A., Adjei, K., Apusiga, K., Gyamfi, J., Asante, K. P., and Plange-Rhule, J. (2019). Capacity and readiness for implementing evidence-based task-strengthening strategies for hypertension control in ghana: A cross-sectional study. *Global heart*, 14(2):129–134.

OECD (2016). *OECD Reviews of Health Systems: Mexico 2016*.

OECD (2020). Tackling the obesity epidemic in mexico 2020.

Prince, M. J., Ebrahim, S., Acosta, D., Ferri, C. P., Guerra, M., Huang, Y., Jacob, K., Jimenez-Velazquez, I. Z., Rodriguez, J. L., Salas, A., et al. (2012). Hypertension prevalence, awareness, treatment and control among older people in latin america, india and china: a 10/66 cross-sectional population-based survey. *Journal of hypertension*, 30(1):177–187.

Rehm, J. and Ferreira-Borges, C. (2018). Risk factor policies, morbidity, and mortality in russia. *The Lancet*, 392(10153):1094–1095.

Rodríguez, E. C., de la Torre Ruiz, H. A., Dávila, S. O. R., et al. (2016). Características y determinantes de la informalidad laboral en méxico. Technical report, Cuerpo Académico 41 de la Universidad Autónoma de Ciudad Juárez.

Rotar, O., Konradi, A., Tanicheva, A., Nakonechnikov, S., Blinova, N., Beaney, T., Xia, X., Poulter, N. R., Chazova, I., and Shlyakhto, E. (2019). May measurement month 2017 in russia: hypertension treatment and control—europe. *European Heart Journal Supplements*, 21(Supplement_D):D101–D103.

Sheiman, I., Shishkin, S., and Shevsky, V. (2018). The evolving semashko model of primary health care: the case of the russian federation. *Risk management and healthcare policy*, 11:209.

Thomas, R., Burger, R., and Hauck, K. (2018). Richer, wiser and in better health? the socioeconomic gradient in hypertension prevalence, unawareness and control in south africa. *Social Science & Medicine*, 217:18–30.

Thorogood, M., Goudge, J., Kabudula, C. W., Limbani, F., Roseleur, J., and Gómez-Olivé, F. X. (2019). Time to review policy on screening for, and managing, hypertension in south africa: Evidence from primary care. *PloS one*, 14(1).

Torres, C. G. (2019). Country report: Colombia–approach to healthcare financing in colombia and its impact on quality, affordability and competition. In *The Law and Policy of Healthcare Financing*. Edward Elgar Publishing.

Wang, J.-G. (2019). Why is the chinese hypertension guideline necessary? *Journal of geriatric cardiology: JGC*, 16(3):173.

Wang, Z., Chen, Z., Zhang, L., Wang, X., Hao, G., Zhang, Z., Shao, L., Tian, Y., Dong, Y., Zheng, C., et al. (2018). Status of hypertension in china: results from the china hypertension survey, 2012–2015. *Circulation*, 137(22):2344–2356.

Zheng, X., Xiao, F., Li, R., Yin, D., Xin, Q., Yang, H., Yin, T., Wang, L., and Chen, B. (2019). The effectiveness of hypertension management in china: a community-based intervention study. *Primary Health Care Research & Development*, 20.
