# Supplementary material for: Wealth and inequality gradients for the detection and control of hypertension in older individuals in middle-income economies around 2007-2015
Source: PLoS One. 2022 Jul 8;17(7):e0269118. doi: 10.1371/journal.pone.0269118 (PMC9269405; doi:10.1371/journal.pone.0269118)
Supplement: Attachment — Submitted filename: PONE-D-21-31685.pdf [file pone.0269118.s002.pdf]

PLOS ONE

Wealth and inequality gradients for the detection and control of hypertension in older individuals in middle-income economies

--Manuscript Draft--

**Manuscript Number:**	PONE-D-21-31685
**Article Type:**	Research Article
**Full Title:**	Wealth and inequality gradients for the detection and control of hypertension in older individuals in middle-income economies
**Short Title:**	Wealth and inequality gradients of hypertension in older individuals in middle-income economies
**Corresponding Author:**	Paul Rodríguez-Lesmes, Ph.D.<br>Universidad Del Rosario<br>Bogota, COLOMBIA
**Keywords:**	Hypertension; Objective health; Self-reported health; Wealth inequalities; health behaviours
**Abstract:**	Socioeconomic inequalities in the detection and treatment of non-communicable diseases represent a challenge for healthcare systems in middle-income countries (MICs) in the context of population ageing. This challenge is particularly pressing regarding hypertension due to its increasing prevalence among older individuals in MICs, especially among those with lower socioeconomic status (SES). Using comparative data for China, Colombia, Ghana, India, Mexico, Russia and South Africa, we systematically assess the association between SES, measured in the form of a wealth index, and hypertension detection and control. Furthermore, we determine what observable factors, such as socio-demographic and health characteristics, explain existing SES-related inequalities in hypertension detection and control using a Blinder-Oaxaca decomposition. Results show that the prevalence of undetected hypertension is significantly associated with lower SES. For uncontrolled hypertension, there is evidence of a significant gradient in three of the six countries. Differences between rural and urban areas as well as lower and higher educated individuals account for the largest proportion of SES-inequalities in hypertension detection and control. Improving access to detection and treatment of hypertension in rural areas and among lower educated individuals may lead to sizeable reductions in wealth-related inequalities in hypertension in MICs.
**Order of Authors:**	Maria Fernanda Garcia
	Philipp Hessel
	Paul Rodríguez-Lesmes, Ph.D.
**Additional Information:**	
**Question**	**Response**
**Financial Disclosure**<br><br>Enter a financial disclosure statement that describes the sources of funding for the work included in this submission. Review the submission guidelines for detailed requirements. View published research articles from *PLOS ONE* for specific examples.<br><br>This statement is required for submission and **will appear in the published article** if the submission is accepted. Please make	GADC project by the CIHR/IDRC [grant number 108442-001]<br>Fulbright-Colciencias and Colombia Cientifica – Alianza EFI 60185 contract FP44842-220-2018, funded by The World Bank through the Scientific Ecosystems, managed by the Colombian Ministry of Science, Technology and Innovation (MINCIENCIAS)

Powered by Editorial Manager® and ProduXion Manager® from Aries Systems Corporation

sure it is accurate.<br><br>**Unfunded studies**<br>Enter: *The author(s) received no specific funding for this work.*<br><br>**Funded studies**<br>Enter a statement with the following details:<br>• Initials of the authors who received each award<br>• Grant numbers awarded to each author<br>• The full name of each funder<br>• URL of each funder website<br>• Did the sponsors or funders play any role in the study design, data collection and analysis, decision to publish, or preparation of the manuscript?<br>• **NO** - Include this sentence at the end of your statement: *The funders had no role in study design, data collection and analysis, decision to publish, or preparation of the manuscript.*<br>• **YES** - Specify the role(s) played.<br><br>* typeset	
**Competing Interests**<br><br>Use the instructions below to enter a competing interest statement for this submission. On behalf of all authors, disclose any competing interests that could be perceived to bias this work—acknowledging all financial support and any other relevant financial or non-financial competing interests.<br><br>This statement is required for submission and **will appear in the published article** if the submission is accepted. Please make sure it is accurate and that any funding sources listed in your Funding Information later in the submission form are also declared in your Financial Disclosure statement.<br><br>View published research articles from *PLOS ONE* for specific examples.	The authors have declared that no competing interests exist.

**NO authors have competing interests**<br><br>Enter: *The authors have declared that no competing interests exist.*<br><br>**Authors with competing interests**<br><br>Enter competing interest details beginning with this statement:<br><br>*I have read the journal's policy and the authors of this manuscript have the following competing interests: [insert competing interests here]*<br><br>* typeset	
**Ethics Statement**<br><br>Enter an ethics statement for this submission. This statement is required if the study involved:<br><br>• Human participants<br>• Human specimens or tissue<br>• Vertebrate animals or cephalopods<br>• Vertebrate embryos or tissues<br>• Field research<br><br>Write "N/A" if the submission does not require an ethics statement.<br><br>General guidance is provided below. Consult the submission guidelines for detailed instructions. **Make sure that all information entered here is included in the Methods section of the manuscript.**	N/A

Powered by Editorial Manager® and ProduXion Manager® from Aries Systems Corporation

**Format for specific study types**<br><br>**Human Subject Research (involving human participants and/or tissue)**<br>• Give the name of the institutional review board or ethics committee that approved the study<br>• Include the approval number and/or a statement indicating approval of this research<br>• Indicate the form of consent obtained (written/oral) or the reason that consent was not obtained (e.g. the data were analyzed anonymously)<br><br>**Animal Research (involving vertebrate animals, embryos or tissues)**<br>• Provide the name of the Institutional Animal Care and Use Committee (IACUC) or other relevant ethics board that reviewed the study protocol, and indicate whether they approved this research or granted a formal waiver of ethical approval<br>• Include an approval number if one was obtained<br>• If the study involved *non-human primates*, add *additional details* about animal welfare and steps taken to ameliorate suffering<br>• If anesthesia, euthanasia, or any kind of animal sacrifice is part of the study, include briefly which substances and/or methods were applied<br><br>**Field Research**<br><br>Include the following details if this study involves the collection of plant, animal, or other materials from a natural setting:<br>• Field permit number<br>• Name of the institution or relevant body that granted permission	
**Data Availability**<br><br>Authors are required to make all data underlying the findings described fully available, without restriction, and from the time of publication. PLOS allows rare exceptions to address legal and ethical concerns. See the PLOS Data Policy and FAQ for detailed information.	Yes - all data are fully available without restriction

Powered by Editorial Manager® and ProduXion Manager® from Aries Systems Corporation

A Data Availability Statement describing where the data can be found is required at submission. Your answers to this question constitute the Data Availability Statement and **will be published in the article**, if accepted.<br><br>**Important:** Stating 'data available on request from the author' is not sufficient. If your data are only available upon request, select 'No' for the first question and explain your exceptional situation in the text box.<br><br>Do the authors confirm that all data underlying the findings described in their manuscript are fully available without restriction?	
**Describe where the data may be found in full sentences. If you are copying our sample text, replace any instances of XXX with the appropriate details.**<br><br>• If the data are **held or will be held in a public repository**, include URLs, accession numbers or DOIs. If this information will only be available after acceptance, indicate this by ticking the box below. For example: *All XXX files are available from the XXX database (accession number(s) XXX, XXX.*).<br>• If the data are all contained **within the manuscript and/or Supporting Information files**, enter the following: *All relevant data are within the manuscript and its Supporting Information files.*<br>• If neither of these applies but you are able to provide **details of access elsewhere**, with or without limitations, please do so. For example:<br><br>*Data cannot be shared publicly because of [XXX]. Data are available from the XXX Institutional Data Access / Ethics Committee (contact via XXX) for researchers who meet the criteria for access to confidential data.*<br><br>*The data underlying the results presented in the study are available from (include the name of the third party*	Data is available at the SAGE project webpage:<br>https://apps.who.int/healthinfo/systems/surveydata/index.php/catalog/sage/about<br>And upon request by the Ministry of Health of Colombia

Powered by Editorial Manager® and ProduXion Manager® from Aries Systems Corporation

and contact information or URL).<br>• This text is appropriate if the data are owned by a third party and authors do not have permission to share the data.<br><br>* typeset	
Additional data availability information:	

Manuscript

Click here to access/download;Manuscript;Ageing_HBP_Control_LMIC.pdf

Wealth and inequality gradients for the detection and control of hypertension in older individuals in middle-income economies

María Fernanda García[1], Philipp Hessel[2], Paul Rodríguez-Lesmes[1¤]

1 School of Economics, Universidad del Rosario. Bogotá, Colombia.
2 Escuela de Gobierno, Universidad de los Andes. Bogotá, Colombia.

¤Corresponding author: paul.rodriguez@urosario.edu.co
Calle 12 C No. 4 - 69; Casa Pedro Fermín de Vargas 111711; Bogotá, Colombia.

Abstract

Socioeconomic inequalities in the detection and treatment of non-communicable diseases represent a challenge for healthcare systems in middle-income countries (MICs) in the context of population ageing. This challenge is particularly pressing regarding hypertension due to its increasing prevalence among older individuals in MICs, especially among those with lower socioeconomic status (SES). Using comparative data for China, Colombia, Ghana, India, Mexico, Russia and South Africa, we systematically assess the association between SES, measured in the form of a wealth index, and hypertension detection and control. Furthermore, we determine what observable factors, such as socio-demographic and health characteristics, explain existing SES-related inequalities in hypertension detection and control using a Blinder-Oaxaca decomposition. Results show that the prevalence of undetected hypertension is significantly associated with lower SES. For uncontrolled hypertension, there is evidence of a significant gradient in three of the six countries. Differences between rural and urban areas as well as lower and higher educated individuals account for the largest proportion of SES-inequalities in hypertension detection and control. Improving access to detection and treatment of hypertension in rural areas and among lower educated individuals may lead to sizeable reductions in wealth-related inequalities in hypertension in MICs.

1 Introduction

The burden of non-communicable diseases (NCDs) in middle-income countries (MICs) is growing due to demographic and lifestyle changes. Already 85% of all premature NCDs deaths are in MICs (1). This is a pressing concern for low-resourced economies which have yet to deal with transmissible diseases as well as the lack of adequate human capital and physical resources of their healthcare systems (2), therefore threatening to achieve the Sustainable Development Goal of universal healthcare coverage that most countries are committed to (3). One condition that summarises this threat is hypertension or high blood pressure (HBP), a condition which affects 22% of the population aged 18 years or older (1) and about 60% of individuals aged 60 years or older worldwide (4). Yet, it is preventable and treatable, making it a specifically useful measure of healthcare system effectiveness (5).

Besides universal coverage, another critical objective of healthcare systems is to improve health equity, thus reducing inequalities in health according to socioeconomic

status (SES), and in particular to wealth (6). There exist significant health disparities according to SES in most MICs, with an epidemiological transition occurring during which the disease burden of NCDs is shifting from individuals with higher SES to individuals with lower SES (7). Inequalities in access are often associated with the source of health insurance funding, partly driven by the large informal sector of the economy in MICs.

Although the successful prevention and treatment of NCDs among individuals from all socio-economic backgrounds present a litmus test for health systems in MICs (8), evidence on inequalities in NCDs in MICs and the potential effectiveness of healthcare systems in reducing the latter has at least two crucial shortcomings. First, few studies use objectively measured prevalence of NCD in general and HBP in particular, mostly relying on self-reported prevalence. This is problematic for two reasons. On the one hand, as a result of often poor health literacy among individuals with low SES, self-reported prevalence often overestimate their health status and are thus not aware of existing health conditions (9). On the other hand, self-reports of HBP prevalence mostly relies on questions asking about conditions diagnosed by a doctor. This may lead to significant under-estimations of HBP prevalence among individuals from lower SES groups due to well-documented barriers in access to healthcare services among these groups. Second, although some studies have assessed the magnitude of inequalities in objectively measured HBP in MICs (10; 11; 12; 13; 14; 15; 16; 17; 18; 19; 20; 21), little evidence exists on the factors associated with those inequalities, and especially the question whether healthcare systems effectively reduce inequities in health.

In this study, we use information from a group of seven MICs (China, Colombia, Ghana, India, Mexico, Russia and South Africa) to assess: 1) levels of SES-related inequalities in (undetected and uncontrolled) HBP, 2) the association between HBP with individuals' socio-demographic characteristics as well as health insurance coverage, and 3) the contribution of different socio-demographic characteristics in explaining existing SES inequalities in HBP.

While undetected and uncontrolled HBP represent two inter-related outcomes of great importance for assessing wealth-related inequalities, given their direct relevance for HBP prevalence in the population, several mechanisms may explain SES-related inequalities therein. On the one hand, differences in (access to) material as well as immaterial resources, including income, transportation, time and health literacy between wealth groups, likely leads to individuals with lower SES having lower chances of being successfully diagnosed with HBP as well as having controlled HBP compared to individuals with higher wealth. For example, individuals with lower SES may lack the income to pay for transportation to see a doctor, while - at the same time - may also face significantly longer transportation times to the nearest doctor, compared to individuals with higher wealth. On the other hand, features of the healthcare system may exacerbate such SES-related inequalities in various ways. For example, lack of health insurance and co-payments for doctor visits and medicines often represent a significant barrier for effective healthcare coverage among individuals with lower wealth. Compared with individuals with higher SES, individuals with lower wealth are therefore less likely to consult a doctor, i.e., be diagnosed with HBP and less likely to be successfully treated for HBP. For the aforementioned reasons, we hypothesise that - in all countries under study - lower SES is significantly associated with a lower likelihood of detected and controlled HBP.

2 Materials and Methods

2.1 Data

The Study on Global Ageing and Adult Health (SAGE) project compiles studies on the population aged 60 and older. Countries included are China, Ghana, India, Mexico, Russian Federation and South Africa. We are using SAGE wave 0, which for China includes information for years 2007/08 and 2009/10; Ghana 2007/08; India 2007/08; México 2009/10; Russia 2007/08 and 2009/10; and South Africa 2007/08 (22).[1] These surveys measure the determinants of active ageing through SES data, the physical and social environment, behaviour, cognition and affect, functionality, mental well-being, medical and health conditions, as well as use and access to health services.

For Colombia, the Healthcare, Welfare and Ageing Survey (SABE, for its acronym in Spanish) was collected in 2015, and its design is comparable to the other ageing population surveys (23). Appendix A presents the main characteristics of the health systems of these countries.

One of the main characteristics of those surveys among older individuals is that they involve clinical measures such as blood pressure. Following a standardised procedure, nurses measured three times systolic and diastolic BP. After discarding the first BP measurement, we take as our objective measure of HBP whether the average of the measurements is above 140 mmHg for systolic BP, or above 90 mmHg for diastolic BP.[2]

We include in our analysis all observations of individuals aged 60 and older for whom there is available information in the following characteristics:[3] valid measurements of blood pressure, self-reported diagnosis of high blood pressure, gender, age, education, weight and height, smoking behaviour, assets, and health insurance (in all countries but Mexico). As a common limitation for using biomarkers data, the resulting sample is likely to be more educated, wealthier and care more for their health than the regular population (24; 25).[4] Table 1 presents the means of the variables included, and sample sizes. In the rest of the document we explain the table and how variables are used. Individual sample weights are used in all exercises.[5]

2.2 High blood pressure classification

Following (26), we classify respondents in four groups, based on whether they are aware of having HBP and objective BP measures. Someone is considered **aware** if he or she reports having been diagnosed with HBP by a doctor or nurse, excluding women diagnosed during pregnancy. The groups are defined as follows. (i) **No HBP:** those respondents who declare not having being diagnosed with HBP and do not present objectively measured BP levels that indicate otherwise. (ii) **Undetected HBP:** those respondents who report not having HBP, but according to their BP measurements, they

[1]Unfortunately, at the time of writing this study, we were not able to get access to the second wave studies collected at 2014/15.

[2]This is a standard procedure as the first BP measurement is usually higher than average because respondents tend to be nervous, a phenomenon known as the *white-coat effect*. In SABE, systolic BP was measured in both arms (six measurements), while in SAGE it only was in one arm (three measurements). In Mexico, there were only two measurements, so we take the second one. Figure A1 in the appendix presents the densities of the final systolic BP measure for each country. In this set of six countries, we observe an ample range of BP patterns: from the low levels of India to the high ones of South Africa.

[3]SAGE studies include information for individuals 50 to 59 as well. We exclude them as SABE does not cover this age group. Nevertheless, online appendix D presents the exercise using this sample, which are qualitatively the same as those presented in the main text.

[4]See appendix B for further details, and the limitations under the discussion section for the implications of the selection pattern.

[5]These weights account for sample selection. Further details are available at: `https://www.who.int/data/data-collection-tools/study-on-global-ageing-and-adult-health/sage-waves`

likely have the condition. (iii) **Uncontrolled HBP:** The person is *aware* of being diagnosed with HBP, but the BP measurements suggest that current treatment (if any) is ineffective. And (iv) **Controlled HBP:** those respondents who are *aware* of HBP, and whose BP levels are under control.

In this study, the denominator of our analysis are individuals who either report having ever been with HBP, or those whose BP is above the standard diagnosis threshold (groups ii to iv). Henceforth, we refer to them as the **at-risk population (AR)**. While group (i) is essential for public health interventions to prevent them from developing HBP, in this work, we concentrate on those who already require attention by the health system. In this sub-population, it is possible to discuss the false negatives of the system (group ii).[6] The other leading group of interest is (iii), as it involves those for whom the health system has not provided effective treatment. Ideally, these two groups should be zero. Another element of interest is the proportion of those who are aware (groups iii and iv) and are receiving treatment. We concentrate on an outcomes perspective, rather than on an input perspective. Therefore we make emphasis in the controlled/uncontrolled partition. Thus, our main dependent variables will be:

- to have undetected HBP for those individuals in the population AR;
- to have uncontrolled HBP conditional on being aware of the HBP condition.

2.3 Socio-economic status / Wealth index

In order to understand the role of SES on our HBP variables, we constructed a **wealth index** based on assets' ownership. This is a composite measure of a household's cumulative living standard. This index measures wealth based on household's ownership of selected assets and is built using factor analysis. Details of the weights given to each variable are presented in the online appendix B. The index was constructed with the following variables: the household appliances that counted the home, type of dwelling, housing rights (ex: own house, rent), physical characteristics of the dwelling (roofing, walls and floors material). In the Colombian case, we also included a socio-economic level indicator known as *estrato* (according to a utility subsidies and taxes classification). Figure 1 presents estimated densities of the index for each country. The value of the index is relative for each country: our objective is to understand the concentration of wealth, rather than it's level. As shown in the graph, India and Ghana are in one extreme, while Russia is in the other. All the other countries are relatively similar in that respect.

2.4 Discrete choice models

In order to estimate the relationship between undetected and uncontrolled HBP with household wealth, we estimate logistic regression models. The probability of observing that for individual i outcome Y_i take the value of 1 instead of 0, conditional on a set of covariates X_i, country R_i, and wealth index $wealth_i$ is given by the following equation:

$$Pr(Y_i = 1|X_i) = \Lambda\left(\sum_{r=1}^{6}(\alpha_r + \gamma_r \cdot \text{wealth}_i + X_i\beta_r)\cdot 1[R_i = r]\right) \quad (1)$$

where Λ represents the logistic function. For the case of uncontrolled HBP, the sample is restricted to those diagnosed with the condition. In this regression, on top of

[6] False-positives, those diagnosed with HBP but who did not have the condition, cannot be detected with this survey-based approach. This group is important as it indicates the inefficient use of resources of the system.

the countries fixed effects and a dummy for respondents being surveyed in 2009/10,[7] we include the interaction between the country dummies and the independent variables. As a result, the interaction of the wealth index and the country dummy provides a measure of the gradient (γ_r) for each country. These models were estimated using Stata 16.

From the results of these models, we compute the average marginal effects (AME) of wealth on the respective probabilities: $E\left[\frac{\partial Pr(Y_i=1|X_i,R_i)}{\partial wealth_i}\right]$. This expectation can be computed conditional on the country. The resulting figures can be interpreted as increases on percentage points (pp) over the probability of $Y = 1$ as opposite to $Y = 0$. The odds ratio, an alternative for interpreting the coefficients of the model, are presented in the appendix table C1.

A generalisation of the model is the multinomial logistic model. This model fits the context as our at-risk group is composed of three categories. The model considers the probability of occurrence of two of the three events relative to a basis. We present in the appendix table C1 the relative risk ratios (rrr), similar to the odds ratio in the binary case.

2.5 Blinder Oaxaca decomposition

This decomposition technique was used to study mean outcome differences in uncontrolled and undetected High blood pressure (HBP) by poverty status. We considered as poor to 20% of people with the lower income.[8] This method divides the uncontrolled or undetected HBP differential between two groups (poor and non-poor) into a part that could be explained by group observable characteristic differences and a residual part that cannot be accounted for by such differences in HBP determinants. The unexplained part subsumes the effects of group differences in unobserved predictors.

We used a twofold decomposition using the coefficients from a pooled linear probability model over both groups as the reference coefficients. Then, the outcome difference is divided into two components:

$$R = Q + U \tag{2}$$

where the first component,

$$Q = \{E(X_{poor}) - E(X_{nonpoor})\}'\beta^* \tag{3}$$

is the part of the outcome differential that is explained by group differences in the predictors. As predictors, we used behavioural risks which include the variables of age and being male, demographic risks, which have smoking history and obesity status, and a variable that indicates if the individual lives in an urban area. The second component in this decomposition,

$$U = E(X_{poor})'(\beta_{poor} - \beta^*) + E(X_{nonpoor})'(\beta^* - \beta_{nonpoor}) \tag{4}$$

is the unexplained part, which in addition to capture all the potential effects of differences in unobserved variables can be a proxy of inequality in HBP by poverty status.

(27) consider an extension of the original decomposition but using non-linear probability models such as the logit and probit. Under this framework, the contribution of each variable to the gap is equivalent to the change in the average predicted probability if the distribution of the poor is replaced with the distribution of the nonpoor. According to the authors, the linear probability model may produce

[7] The comparison group is being surveyed on 2007/08. The Colombian survey was the only one in 2015, so its information is already part of the specific survey fixed effect.

[8] Sensitivity to alternative cutoffs is presented in the online appendix G.

notoriously different covariates that have strongly non-linear relationships with the outcome. Therefore, we compute the decomposition under this extension in the appendix C. These models were estimated using the command Oaxaca in Stata 16 (28).

3 Results

3.1 High Blood Pressure groups and wealth

Panel A of Table 1 presents the means of the relevant variables conditional on different samples (for which there is no missing data in all covariates). Columns marked with BPS (Blood Pressure Sample), corresponds to those individuals for whom there is information on their blood pressure. Given that, we can determine the proportion of total respondents who are AR, which is presented in the first row. In Russia, South Africa, around 81% of respondents aged 60 or older are AR, followed by Mexico (77%) and Colombia (69%). Below is China (67%), and far from them are Ghana (57%) and India (39%). The second row presents those who are aware of having HBP. If the denominator is the entire population (columns BPS), we have the detected prevalence, which is as high as 61% in Russia and as low as 15% in Ghana.

The other set of columns of 1, present the proportions for the population AR. For instance, China has a detected prevalence of 33% on the overall 60 and older population. These individuals are nearly half (49%) of those who are at risk, the AR population. Thus, the undetected BP rate is 51%. Relative to the AR population, 12% have their BP levels controlled (systolic/diastolic BP below 140/90 mmHg). Thus, the proportion of individuals aware, but with their BP being uncontrolled, corresponds to 39% (=51% - 12%) of the AR population. This is is the uncontrolled HBP rate. Panel B of table 1 presents the total number of observations for each of these groups.

Concerning undetected HBP there are four groups. First, in Colombia and Russia, undetected HBP rates are 18% and 25%, respectively. Second, for Mexico is 45%. Third, for China, India, and South Africa is around 53%. And fourth, in Ghana, this figure is just above 74%. Once we consider control of the condition, the best work is for India: 20% of Indians AR have their systolic BP below 140 mmHg and their diastolic BP below 90 mmHg. The worst case is Russia, where 61% of the AR are known cases of HBP which are not under control.

Table 1 presents as well socio-economic characteristics of the respondents. Age and gender produce 'mechanical' differences between countries. However, Russia has fewer men in the sample (33%), yet BP levels are one of the highest; the South African population is on average as old as the Indian (68 years), but there are more than ten mmHg of difference between their BP levels. Differences are more likely to be related to habits and lifestyle: obesity in South Africa is nearly 50%, while in India it is 4%. Yet, China obesity rate is as well low, 7%, but their average systolic BP is the same in South Africa. Mexico and Colombia have the highest rate of people living in an urban area, close to 80%. However, BP in Mexico is higher than in Colombia. Although Mexico has the highest wealth index also has one of the highest BP in our sample of countries. Another consideration is that there are no substantial systematic differences between the BPS and AR population because AR represents a large proportion of all individuals older than 60 in these countries.

Another set of variables presented in Table 1 is related to the health system. As described in the context section, there are noteworthy differences. Russia has no VHI at all, while in Ghana, 41% of the individuals have it. Almost the rest of the population is uninsured in Ghana, only worse to India, where nine out ten did not have such protection. The opposite is again Russia, where everyone is insured, similar to Colombia, and where people without health insurance are just 2%. A very similar pattern occurs

for living in rural areas, where more barriers have been found to expand universal coverage in the world. Unfortunately, this information is not available for Mexico.

Socio-economic characteristics, lifestyle, and health system differences shape the relationship between wealth and health in each country. The unconditional relationship between wealth and our HBP quality of attention indicators are presented in Figure 2. Each point of the line presents the average rate of undetected and uncontrolled HBP for each level of wealth. First, it shows a negative slope between wealth and probability of non-detection of HBP, which depends on the country. Second, concerning uncontrolled HBP, there are both positive and negative slopes, showing that the role of wealth is context-dependent. With the AME derived from the parametric model presented above, we can determine whether the observed slopes hold after conditioning of different sets of controls.

3.2 Results from discrete choice models

Table 2 presents the AME after logistic regressions. It considers three models for each outcome which differ on the set of controls. Columns (1) and (5) only consider country fixed effects, age, and a gender dummy. Columns (2) and (6) expand them to lifestyle: whether the individual is obese and if she has ever smoked. These are the main risk factors, and there are not so many differences between both sets of columns. Columns (3) and (7) include education and whether the respondent resides in an urban area. Finally, columns (4) and (8) consider health insurance schemes. These are characteristics that might be correlated with wealth. Thus the resulting coefficients correspond to the remaining disparities not associated with these variables.

If we consider only variables not related to wealth, we find that concerning undetected HBP, there is a wealth gradient in all countries (column 2). Ghana and Russia are the most extreme cases: if wealth has ten levels, moving up in one level is associated with a reduction of 7.27 pp the probability that someone above the age of 60 has undetected hypertension in Ghana, and of 7.64 pp in Russia, conditional on risk factors such as obesity and smoking antecedents. If we consider education level on top of these controls (column 3), the gradient drops to 6.64 pp in Ghana and 7.45pp in Russia. South Africa is on the opposite scenario, where the gradient is around 2.05 pp without education and 1.66 pp (not significant at 90% level) when these controls are added. Health system variables reduce the gradient slightly in most countries, especially in China. The considerable reduction on the gradient for Russia has to be taken carefully, as less than 1% of the individuals at risk has VHI, and none has no insurance. Therefore the change is such point estimate obeys more to the high imprecision of the estimate (a 95% confidence interval between -6.28 pp and -83.11 pp).

Regarding uncontrolled HBP, table 2 presents a similar analysis but conditional on the diagnosis. Here we are exploring the probability of having HBP in comparison with a mix between those people with controlled HBP and those who are not aware of the condition.[9] The table shows a significant negative gradient for all countries but for Mexico and South Africa. For these countries, the gradient is negative as well, but the precision of the estimates cannot rule out the non-presence of an association. The largest figure is for Russia (-0.41 in column 6) and Mexico (-0.42, but not significant at 95% level), and the smallest for South Africa (-0.11). Notice that the dispersion between countries is smaller than for detection. In most cases, the addition of health insurance variables reduces the magnitude of the gradient, especially for China where the gradient disappears.

Estimated odd ratios of the models (3) and (6) are presented in table C1 in the appendix, using the same controls as columns 3 and 7 of table 2, as well as regressions

[9] In the appendix F, we present results without conditioning on the diagnosis.

for systolic blood pressure and an alternative multinomial logistic model. Regardless of the model specification, and even with the continuous systolic BP variable, similar patterns to the ones presented above are obtained. This is also the case when alternative definitions of the age range (appendix D) and the assets index (appendix E); and when individual regressions per country and set of controls are considered (appendix H).

3.3 Decomposition results

We further explore the gradient by comparing differences between those at the bottom of the wealth distribution (the lowest quintile) with the rest of the sample. The black diamonds in Figure 3 present the difference in the proportion of undetected HBP (on the left) and uncontrolled HBP (on the right). Concerning the previous section, the pattern for the overall gap differs in the case of Russia. For this country, there is a small gap in this exercise while a large wealth gradient. This indicates that the gradient is probably linked to a specific upper interval of the domain of the wealth index (where most of the distribution is located as shown in Figure 1), and not a feature that is constant across the wealth distribution. For Ghana, the large gap goes in line with the steep gradient.

For this exercise, we focus on decomposition analysis. For example, for China the gap between *poor* and *rich* on undetected HBP is of -23.3 pp. This number is obtained from taking the undetected HBP rate of 44.9% for the 80% of the sample with higher wealth and subtracting the 68.1% rate for the lower quintile (see Table C2 in the appendix). For this alternative view of the gradient, we can decompose the contribution of each of the observed factors -behaviours, demographics, education and urban/rural division- using the Blinder-Oaxaca method. For the case of China, 56.2%(=-0.131/-0.233) of the gap can be explained with the difference in the level of the observed variables. This is roughly the same proportion for most countries (from 45% to 58.4%, apart from Mexico and South Africa. In Mexico, it is 34.4% of a gap of 7.32 pp which is not significant at the 95% level, and in South Africa, it is 86.32% of the gap of 8.41 pp can be explained with the observed variables.

Almost all the explained differences are due to the urban/rural division in the first place and the education level in the second. Again, for the case of China, differences in the proportion of people living in urban areas represent 80.15%(=-0.105/-0.131) of the explained difference; this figure is also high for South Africa (83.5%), and very small in Mexico (3.4%). Differences in education level represent another 11.8% in China, and just an 8% for the rest of the variables (socio-demographics, smoking prevalence and obesity). In Mexico, 91.3% of all the explained gap can be associated with education. In general, these two factors account for most of the observed gap (more than 80% of it). However, in Ghana, age and gender differences represent nearly 11.3% of the explained difference. In Figure C1 in the appendix, we perform the decomposition separately by urban and rural areas. It shows that all the other observed characteristics play a minor role in explaining wealth gaps within these areas.[10]

Tables C5 in the appendix includes the health insurance variables into the decomposition. The proportion of the gap explained is similar to the results above. These variables explain 14% of the difference in Ghana, 11.6% in South Africa, 10.6% in India, 9.4% in Colombia, and 2.5% in Russia. In contrast, for China (-3.5%) the level of observable variables suggest that the gap should run in the opposite direction.

The absolute difference in uncontrolled HBP, conditional on the diagnosis, between poor and non-poor people is statistically significant for China, Colombia and South Africa (see Table C3 in the appendix). For the other countries, the total difference cannot be rejected to be equal to zero. For Ghana, India, Mexico, and Russia, there is

[10]For Ghana, the number of observations with a positive diagnosis of HBP in the rural setting is too small for the analysis.

no evidence of a gap though there was evidence of a gradient. Once again, this might be related to the point of comparison of the wealth distribution. Ghana presents interesting results, as the point estimate is small, but the explained component suggests a gap similar to the Colombian or the Indian. For this country, any analysis should be careful as the number of individuals diagnosed with HBP is small, so results are very imprecise.

For the three countries for which there is evidence of a gap, the explained component is roughly between 50% and 60%. For China and Colombia, more than 90% is explained by rurality, while this factor is only 36% in South Africa. In the last country, education accounts for 77% of the explained gap, while it is 6% in China and Colombia. When health insurance variables are included (Table C5 in the appendix), there are almost no changes at all, and these variables explain around 4% to 6% of the explained gap.

As discussed in the methods section, the BO decomposition with linear probability models might be biased for covariates with a strong non-linear relationship with the outcome. In the appendix, tables C6 and C7 show the results of (27) extension based on a logit model. Results are almost the same as those presented above. Moreover, as for the results of the marginal effects, we consider alternative definitions of the age range (appendix D), the assets index (appendix E), and the uncontrolled HBP results conditional on being aware of the conditions (appendix E). We also consider the sensitivity of the results to alternative definitions of the non-poor sample (appendix G). Qualitative results are stable across these alternatives.

4 Discussion

In this study, we explored the role of household wealth on the quality of seven MICs' health systems for attending mature patients aged 60 and older with HBP, both aware and not of their condition. This condition, which requires an effective action from several layers on the primary health care system, is central for preventing the onset of cardiovascular diseases.

As presented by (29), treating HBP has four inter-related goals: awareness, treatment, antihypertensive medication adherence and control. Despite differences in health systems, there exist several common elements for attaining these goals: access to health insurance, low prices/co-payments for medication, consistent access to health care. Over these areas, the MICs analysed in this study present important differences:

- The population at risk is between 67% and 81% in China, Colombia, Mexico, Russia and South Africa, compared to the range of 39% - 57% in Ghana and India. This is potentially linked with the higher urbanisation rates of the first countries. Obesity also might play an essential role for Colombia, Mexico and Russia.
- Russia and Colombia have reasonable detection procedures, which allow them to have high awareness rates (75%-82%). Ghana does a poor job with 26%, and in other countries included in this study, only half of those at risk are aware of their condition.

An important indicator of health system effectiveness is the degree to which it reduces wealth-related inequalities in health. As the results of this study show, in all of the MICs included, there are significant wealth-related differences in the likelihood of having detected HBP, with individuals with lower wealth being less likely to be diagnosed than those with higher wealth. To explore the roots of these gradients, we considered the following factors: demographic (age and gender), behavioural (obesity and smoking), socio-economic (education and living in urban areas), and health system (access to compulsory and voluntary health insurance). We found that:

- Wealth is essential in all countries for determining awareness, but it is especially relevant in Ghana and Russia. In all countries, the wealth gradient is less pronounced once we control for access to HI.
- Once diagnosed with HBP, the wealthier the individuals, the more likely their BP is under control. For China, the gradient disappears once health insurance is under control.
- The most relevant observed factor for the gap on undetected and uncontrolled rates is to live in a rural area rather than an urban area. It is substantially more important for most countries than education level, the second determinant in the list. Differences in demographic characteristics and behavioural risks play a small role in explaining large differences in most countries. This is likely because the wealth gap on these variables is relatively small, rather than that these variables are not associated with the outcomes.

Health insurance plays a different role according to the context. In Ghana, South Africa and India, it accounts for around 10% of the explained gap. In such countries, there are large proportions of the population without any health insurance. In contrast, in Russia and China, these variables explain little of the gap and have small uninsured populations and the VHI sector. Colombia is the exception, as it also has a small VHI sector and few people uninsured, but these variables can explain up to 9.4% of the gap. Russia and Colombia provide different versions of universal coverage: the two have high levels of awareness and very different wealth gradients. Colombia has a gradient that cannot be explained with any of the observed factors, and Russia has a sharp gradient that drops dramatically when access to VHI is considered (yet, it is imprecisely estimated).[11] The reasons behind such divergence need further research to be fully understood.

For two countries, we can present a comparison with the literature. First, for China, the gradient in prevalence is non-existent, and that there is a positive gradient between detection and control (18). Second, for South Africa, results are in line with (11) who found no socio-economic gradient in unawareness once controls are considered. They use a large sample that involves respondents of all ages and estimate the gradients using a finite-mixture model that accommodates unobserved heterogeneity.

For the case of keeping under control the BP of patients who are aware of their condition, wealth gradients are negative as well. Interestingly, its magnitude is the same even for countries such as Colombia and Russia, which have large awareness of the condition. A potential reason is a composition effect: countries first have to detect HBP, but it becomes increasingly complicated to treat the 'marginal' patient. This composition effect is one of the explanations given by (30) for persistent health inequalities in Nordic countries. Despite their generous welfare states, poorer individuals in rich societies usually engage in behaviours linked to bad health due to a persistent difference in immaterial resources. For instance, preferences for unhealthy habits, physical activities, nutrition, etc. The behavioural component does not play a prominent role in our estimates, which probably highlights a limitation of the available measurements for capturing differences in lifestyle. For the rest of the countries, where large proportions of the population aged 60 and older are under risk, gradients are either non-existent or not pronounced enough to be detected. Keeping under control BP of detected individuals is not a trivial problem as it goes beyond the system's coverage. Individual behaviour plays a central role. Patients might improve -or not- their

[11] Another difference is the period: Russia surveys are from 2007 to 2010, while the Colombian one from 2015. However, our comparison is between the state of two health systems based on control of HBP. There are no many differences in terms of the technology available or their prices between these two periods.

health-related behaviour to early detection of the condition and its treatment (31; 32; 33).

It is important to mention that the results presented are associations; they cannot be understood as causal effects between wealth and health. In other words, it cannot be concluded that increasing the wealth of the *current* population will result in smaller probabilities of detection of HBP. As seen in the decomposition, there is a large share of the gap explained by other contextual characteristics related to both variables (in particular, place of residence and education). Also, poor health (which might be correlated with HBP onset) might result in poverty. Exercises that assess the impact of wealth on health require specific exogenous shocks. A good example is (34), who using the reunification of Germany, found only a small impact of income on health satisfaction.

5 Acknowledgements

This project was funded under the GADC project by the CIHR/IDRC [grant number 108442-001], and the program Fulbright-Colciencias and Colombia Cientifica – Alianza EFI 60185 contract FP44842- 220-2018, funded by The World Bank through the Scientific Ecosystems, managed by the Colombian Ministry of Science, Technology and Innovation (MINCIENCIAS).

References

1. WHO. Noncommunicable diseases country profiles 2018. WHO/NMH/NVI/15.1. World Health Organization; 2018.
2. Kassa M, Grace J. The Global Burden and Perspectives on Non-communicable Diseases (NCDs) and the Prevention, Data Availability and Systems Approach of NCDs in Low-resource Countries. In: Non-communicable Diseases and Urbanization-A Global Perspective. IntechOpen; 2019.
3. Desa U, et al. Transforming our world: The 2030 agenda for sustainable development. 2016;.
4. Oliveros E, Patel H, Kyung S, Fugar S, Goldberg A, Madan N, et al. Hypertension in older adults: Assessment, management, and challenges. Clinical Cardiology. 2020;43(2):99–107.
5. Horton S, Gelband H, Jamison D, Levin C, Nugent R, Watkins D. Ranking 93 health interventions for low-and middle-income countries by cost-effectiveness. PLoS One. 2017;12(8):e0182951.
6. Jacobs M, El-Sadr WM. Health systems and health equity: The challenge of the decade. Global Public Health. 2012;1(6):S63–S72.
7. Stringhini S, Bovet P. Socioeconomic status and risk factors for non-communicable diseases in low-income and lower-middle-income countries. The Lancet Global Health. 2017;5(3):e230–e231.
8. Samb B, Desai N, Nishtar S, Mendis S, Bekedam H, Wright A, et al. Prevention and management of chronic disease: a litmus test for health-systems strengthening in low-income and middle-income countries. The Lancet. 2010;376(9754):1785–1797.
9. Johnston DW, Propper C, Shields MA. Comparing subjective and objective measures of health: Evidence from hypertension for the income/health gradient. Journal of health economics. 2009;28(3):540–552.
10. Hessel P, Rodríguez-Lesmes P, Torres D. Socio-economic inequalities in high blood pressure and additional risk factors for cardiovascular disease among older individuals in Colombia: Results from a nationally representative study. PLoS One. 2020;15(6):e0234326.
11. Thomas R, Burger R, Hauck K. Richer, wiser and in better health? The socioeconomic gradient in hypertension prevalence, unawareness and control in South Africa. Social Science & Medicine. 2018;217:18–30.
12. Lloyd-Sherlock P, Minicuci N, Corso B, Beard J, Chatterji S, Ebrahim S. Diseases of the rich? The social patterning of hypertension in six low-and middle-income countries. The European Journal of Development Research. 2017;29(4):827–842.
13. Lloyd-Sherlock P, Beard J, Minicuci N, Ebrahim S, Chatterji S. Hypertension among older adults in low-and middle-income countries: prevalence, awareness and control. International journal of epidemiology. 2014;43(1):116–128.
14. Di Cesare M, Khang YH, Asaria P, Blakely T, Cowan MJ, Farzadfar F, et al. Inequalities in non-communicable diseases and effective responses. The Lancet. 2013;381(9866):585–597.

15. Murphy GA, Asiki G, Ekoru K, Nsubuga RN, Nakiyingi-Miiro J, Young EH, et al. Sociodemographic distribution of non-communicable disease risk factors in rural Uganda: a cross-sectional study. International journal of epidemiology. 2013;42(6):1740–1753.

16. Chow CK, Teo KK, Rangarajan S, Islam S, Gupta R, Avezum A, et al. Prevalence, awareness, treatment, and control of hypertension in rural and urban communities in high-, middle-, and low-income countries. Jama. 2013;310(9):959–968.

17. Hosseinpoor AR, Bergen N, Mendis S, Harper S, Verdes E, Kunst A, et al. Socioeconomic inequality in the prevalence of noncommunicable diseases in low-and middle-income countries: results from the World Health Survey. BMC public health. 2012;12(1):474.

18. Lei X, Yin N, Zhao Y. Socioeconomic status and chronic diseases: The case of hypertension in China. China Economic Review. 2012;23(1):105–121.

19. Vellakkal S, Subramanian S, Millett C, Basu S, Stuckler D, Ebrahim S. Socioeconomic inequalities in non-communicable diseases prevalence in India: disparities between self-reported diagnoses and standardized measures. PloS one. 2013;8(7):e68219.

20. Rehkopf DH, Dow WH, Rosero-Bixby L. Differences in the association of cardiovascular risk factors with education: a comparison of Costa Rica (CRELES) and the USA (NHANES). Journal of Epidemiology & Community Health. 2010;64(9):821–828.

21. Rosero-Bixby L, Dow WH. Surprising SES Gradients in mortality, health, and biomarkers in a Latin American population of adults. Journals of Gerontology Series B: Psychological Sciences and Social Sciences. 2009;64(1):105–117.

22. Kowal P, Chatterji S, Naidoo N, Biritwum R, Fan W, Lopez Ridaura R, et al. Data resource profile: the World Health Organization Study on global AGEing and adult health (SAGE). International journal of epidemiology. 2012;41(6):1639–1649.

23. Albala C, Lebrão ML, León Díaz EM, Ham-Chande R, Hennis AJ, Palloni A, et al. Encuesta Salud, Bienestar y Envejecimiento (SABE): metodología de la encuesta y perfil de la población estudiada. Revista Panamericana de Salud Pública. 2005;17:307–322.

24. Guyer H, Ofstedal MB, Lessof C, Cox K. The Feasibility of Collecting Physical Measures and Biomarkers in Cross-National Studies. In: Population Association of America 2010 Annual Meeting. Session 129: Demographic Studies Based on Biomarkers; 2010.Available from: http://paa2010.princeton.edu/papers/101058.

25. Guyer H, Ofstedal MB, Lessof C, Cox K. The benefits and challenges of collecting physical measures and biomarkers in cross-national studies. Ann Arbor, MI: Institute for Social Research. 2017;.

26. Falaschetti E, Mindell J, Knott C, Poulter N. Hypertension management in England: a serial cross-sectional study from 1994 to 2011. The Lancet. 2014;383(9932):1912–1919.

27. Fairlie RW. An extension of the Blinder-Oaxaca decomposition technique to logit and probit models. Journal of economic and social measurement. 2005;30(4):305–316.

28. Jann B. The Blinder–Oaxaca decomposition for linear regression models. The Stata Journal. 2008;8(4):453–479.

29. Maimaris W, Paty J, Perel P, Legido-Quigley H, Balabanova D, Nieuwlaat R, et al. The influence of health systems on hypertension awareness, treatment, and control: a systematic literature review. PLoS medicine. 2013;10(7).

30. Mackenbach JP. The persistence of health inequalities in modern welfare states: the explanation of a paradox. Social science & medicine. 2012;75(4):761–769.

31. Rodriguez-Lesmes P. Early diagnosis of chronic conditions and lifestyle modification. Documentos de Trabajo Universidad del Rosario. 2017;(015639).

32. Kaestner R, Darden M, Lakdawalla D. Are investments in disease prevention complements? The case of statins and health behaviors. Journal of Health Economics. 2014;36:151 – 163. doi:http://dx.doi.org/10.1016/j.jhealeco.2014.04.006.

33. Zhao M, Konishi Y, Glewwe P. Does information on health status lead to a healthier lifestyle? Evidence from China on the effect of hypertension diagnosis on food consumption. Journal of health economics. 2013;32(2):367–385.

34. Frijters P, Haisken-DeNew JP, Shields MA. The causal effect of income on health: Evidence from German reunification. Journal of health economics. 2005;24(5):997–1017.

Fig 1. Densities of the Wealth index by country

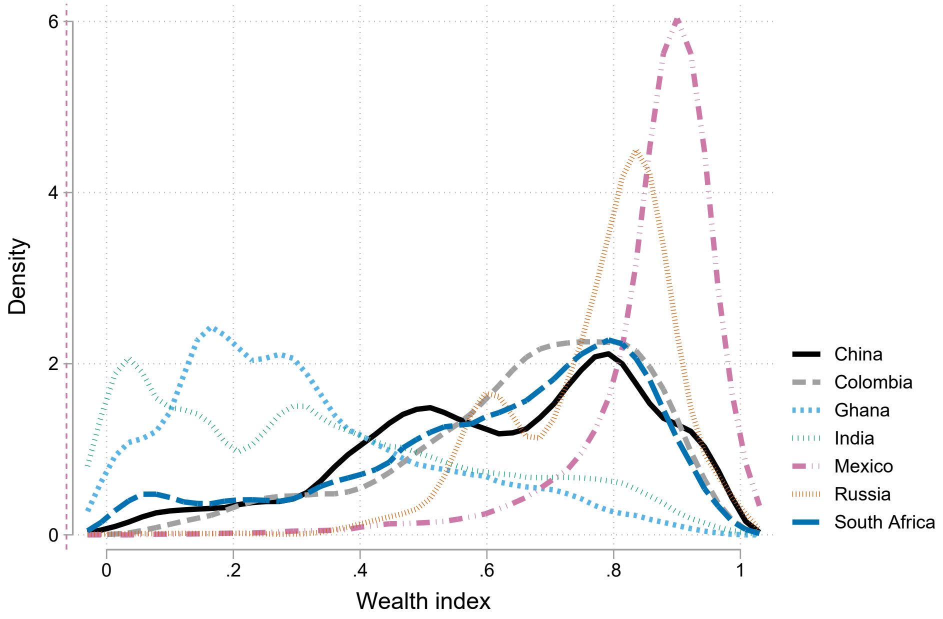

Notes: Epanechnikov Kernel densities using a bandwidth of 0.03 (asset index ranges from 0 to 1). Graph constructed over the sample of SABE and SAGE of respondents with a valid BP measure and a self-report of HBP. The asset index was derived using factor analysis for all individuals who answered the assets section. See online appendix B.2 for further details.

Fig 2. Undetected and uncontrolled HBP and wealth

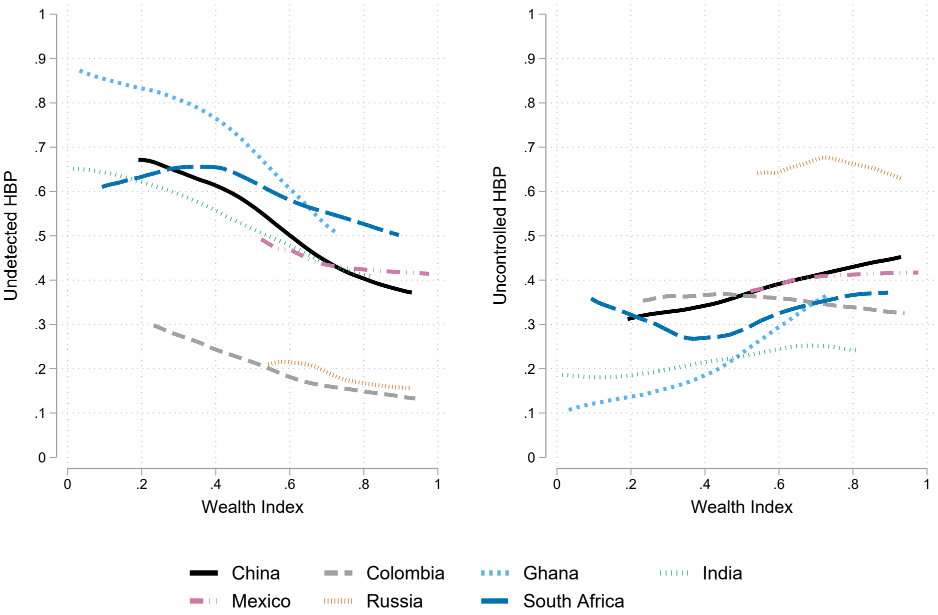

Notes: Kernel-weighted local polynomials using the samples of SABE and SAGE. For each country/region, the domain corresponds to the range covering 5%-95% of the total density of the wealth variable.

Fig 3. Contribution to gap in undetected and uncontrolled HBP conditional on being aware o hypertension

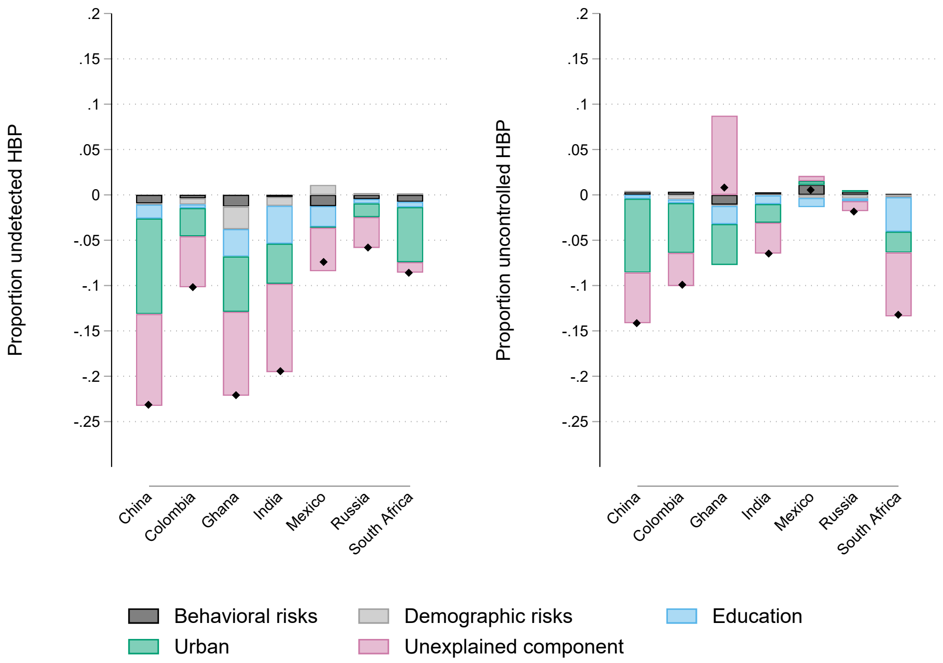

Notes: Contribution of covariate groups to the gap on undetected and uncotrolled HBP between individuals in the lowest wealth quintile agains the other quintiles. These numbers are computed with the Blinder-Oaxaca decomposition after linear probability models.

Table 1. Means by country

Variable	China		Colombia		Ghana		India		Mexico		Russia		South Africa	
	BPS	AR	BPS	AR	BPS	AR	BPS	AR	BPS	AR	BPS	AR	BPS	AR
**A. Proportions and means**														
Population at risk	0.67		0.69		0.57		0.39		0.77		0.81		0.81	
HBP Aware	0.33	0.49	0.57	0.82	0.15	0.26	0.18	0.46	0.43	0.55	0.61	0.75	0.37	0.46
Undetected HBP		0.51		0.18		0.74		0.54		0.45		0.25		0.54
Uncontrolled HBP		0.38		0.35		0.20		0.20		0.38		0.61		0.36
Diastolic BP (mmHg)	83.31	87.30	74.53	76.11	89.88	99.76	80.76	90.94	79.79	82.47	89.80	92.72	95.37	99.29
Systolic BP (mmHg)	145.52	156.28	133.79	140.00	138.65	153.89	124.90	140.79	146.92	154.08	146.24	151.47	148.44	154.59
Male	0.48	0.47	0.43	0.41	0.52	0.50	0.50	0.46	0.47	0.44	0.33	0.31	0.42	0.41
Age	68.94	69.41	70.29	71.20	71.02	70.80	68.19	68.63	69.76	70.06	71.60	72.08	69.00	68.91
Obese (BMI $\geq$ 30)	0.06	0.07	0.33	0.36	0.08	0.11	0.03	0.04	0.27	0.29	0.30	0.33	0.47	0.49
Smoke ever	0.35	0.34	0.51	0.49	0.26	0.23	0.57	0.54	0.44	0.42	0.22	0.19	0.33	0.32
Education: Below Primary	0.49	0.50	0.58	0.59	0.71	0.69	0.66	0.63	0.52	0.53	0.03	0.03	0.51	0.52
Education: Primary	0.22	0.22	0.30	0.30	0.09	0.09	0.16	0.15	0.27	0.27	0.10	0.10	0.22	0.22
Education: Above Primary	0.29	0.28	0.12	0.11	0.20	0.22	0.18	0.22	0.21	0.20	0.87	0.86	0.26	0.25
Lives in urban area	0.51	0.50	0.78	0.79	0.39	0.46	0.29	0.32	0.78	0.80	0.75	0.76	0.64	0.65
Wealth index	0.61	0.61	0.67	0.67	0.33	0.36	0.31	0.36	0.87	0.87	0.78	0.78	0.60	0.61
Voluntary Health Insurance	0.12	0.11	0.06	0.07	0.41	0.41	0.02	0.03			0.01	0.00	0.13	0.13
No health insurance	0.11	0.11	0.02	0.02	0.57	0.56	0.97	0.95			0.00	0.00	0.80	0.80
**B. Number of observations**														
All	6689	4473	5228	3587	2496	1434	3454	1429	1238	893	2271	1827	1958	1572
HBP Aware		2264		2938		358		659		513		1496		677
Undetected HBP		2209		649		1076		770		380		331		895
Uncontrolled HBP		1763		1247		272		307		367		1182		523

Notes: own calculations using SABE and SAGE studies with sample weights. Samples in the columns are defined as follows. **BPS:** those respondents for whom there is information on self-reported HBP and objective measurements. **At risk (AR):** respondents who are either diagnosed with HBP (aware) or not diagnosed but with a systolic BP above 140 mmHg or a diastolic BP above 90 mmHg. The wealth index is defined per country, therefore it cannot be used to compare wealth between countries.

Table 2. Average marginal effects of wealth on undetected and uncontrolled HBP by country

Country	$\frac{\partial \text{Undetected}}{\partial \text{Wealth}}$				$\frac{\partial \text{Uncontrolled}}{\partial \text{Wealth}}$ | Diagnosis = 1			
	(1)	(2)	(3)	(4)	(5)	(6)	(7)	(8)
China	-0.585 ***	-0.558 ***	-0.491 ***	-0.297 ***	-0.343 ***	-0.342 ***	-0.322 ***	0.024
	(0.034)	(0.035)	(0.041)	(0.055)	(0.056)	(0.058)	(0.064)	(0.083)
Colombia	-0.255 ***	-0.255 ***	-0.265 ***	-0.258 ***	-0.257 ***	-0.268 ***	-0.266 ***	-0.166 **
	(0.036)	(0.037)	(0.043)	(0.055)	(0.051)	(0.052)	(0.055)	(0.071)
Ghana	-0.748 ***	-0.727 ***	-0.664 ***	-0.580 ***	-0.303 **	-0.288 **	-0.272 **	-0.244
	(0.059)	(0.063)	(0.074)	(0.099)	(0.135)	(0.131)	(0.126)	(0.180)
India	-0.458 ***	-0.453 ***	-0.308 ***	-0.250 ***	-0.239 **	-0.271 **	-0.296 **	-0.261 *
	(0.062)	(0.065)	(0.072)	(0.090)	(0.107)	(0.117)	(0.124)	(0.142)
Mexico	-0.629 ***	-0.648 ***	-0.694 ***		-0.346	-0.426	-0.192	
	(0.167)	(0.177)	(0.148)		(0.347)	(0.346)	(0.436)	
Russia	-0.821 ***	-0.764 ***	-0.745 ***	-0.447 **	-0.348 **	-0.408 ***	-0.382 ***	-0.441 **
	(0.190)	(0.190)	(0.231)	(0.196)	(0.150)	(0.152)	(0.148)	(0.184)
South Africa	-0.219 **	-0.205 **	-0.166	-0.049	-0.112	-0.115	-0.082	-0.005
	(0.096)	(0.095)	(0.105)	(0.115)	(0.120)	(0.123)	(0.123)	(0.114)
Observations	15127	15127	15127	14223	8767	8767	8767	8247
Age and gender	X	X	X	X	X	X	X	X
Obesity and smoking		X	X	X		X	X	X
Education, urban			X	X			X	X
Health Insurance				X				X

Notes: own calculations using SABE and SAGE studies with individual sample weights. Average marginal effects after logistic regressions are presented in the table. In each estimated equation, each country was multiplied by the wealth index in order to obtain a specific gradient. Controls differ according to columns: (i) age and being male; (ii) obesity status and smoking history; and (iii) education level (primary, and above primary), living in an urban area, having voluntary health insurance, and not having health insurance at all. All regressions include country dummies, a dummy that indicates that the individual was surveyed in year 2009/10 as opposite to 2007/08, and the interaction between each control and this set of dummies. Household level clustered standard errors are presented in parentheses. Significance: * 0.1, ** 0.05, *** 0.01

Click here to access/download
Supporting Information
online_appendix.pdf
