# Supplementary material for: Wealth and inequality gradients for the detection and control of hypertension in older individuals in middle-income economies around 2007-2015
Source: PLoS One. 2022 Jul 8;17(7):e0269118. doi: 10.1371/journal.pone.0269118 (PMC9269405; doi:10.1371/journal.pone.0269118)
Supplement: Attachment — Submitted filename: Comments, PLoS One.docx [file pone.0269118.s003.docx]

**Title of the manuscript:**

Wealth and inequality gradients for the detection and control of hypertension in older individuals in middle-income economies

**Manuscript Number: PONE-D-21-31685**

**Major comments**

(i) The data collection derived from different countries are taken in the basis of the survey using SES data. How can these data can give rise to statistical study? Just surveys can’t be accepted as data.
(ii) The information about the second group in the study population “**Undetected HBP”** is not clear in the methodology. How do you differentiate group 1 **(No HBP)** and group 2 (**Undetected HBP)? Because the definition of both the above groups were similar** (Page no. 3).
(iii) Based on the above comment 2, In the statistical table (Table2), the comparative study between the undetected and uncontrolled HBP is questionable (???).
(iv) In the estimation of relationship between undetected and uncontrolled HBP, the use of the logistic regression model should be justified (Page no. 4).
(v) The Oaxaca (or Blinder-Oaxaca) decomposition usually used to show the differences in an outcome variable across groups or over time can be separated into explained and unexplained portions.

In what way, the above method reflects in this study to find outcome differences in uncontrolled and undetected HBP? It should be justified.
